# Fabrication of monodispersed copper oxide nanoparticles with potential application as antimicrobial agents

**DOI:** 10.1038/s41598-020-73497-z

**Published:** 2020-10-07

**Authors:** Fisseha A. Bezza, Shepherd M. Tichapondwa, Evans M. N. Chirwa

**Affiliations:** Water Utilization and Environmental Engineering Division, Department of Chemical Engineering, University of Pretoria, Pretoria, 0002 South Africa

**Keywords:** Microbiology, Nanoscience and technology

## Abstract

Cuprous oxide nanoparticles (Cu_2_O NPs) were fabricated in reverse micellar templates by using lipopeptidal biosurfactant as a stabilizing agent. Scanning electron microscopy (SEM), transmission electron microscopy (TEM), energy dispersive x-ray spectrum (EDX) and UV–Vis analysis were carried out to investigate the morphology, size, composition and stability of the nanoparticles synthesized. The antibacterial activity of the as-synthesized Cu_2_O NPs was evaluated against Gram-positive *B. subtilis* CN2 and Gram-negative *P. aeruginosa* CB1 strains, based on cell viability, zone of inhibition and minimal inhibitory concentration (MIC) indices. The lipopeptide stabilized Cu_2_O NPs with an ultra-small size of 30 ± 2 nm diameter exhibited potent antimicrobial activity against both Gram-positive and Gram-negative bacteria with a minimum inhibitory concentration of 62.5 µg/mL at pH5. MTT cell viability assay displayed a median inhibition concentration (IC_50_) of 21.21 μg/L and 18.65 μg/mL for *P. aeruginosa* and *B. subtilis* strains respectively. Flow cytometric quantification of intracellular reactive oxygen species (ROS) using 2,7-dichlorodihydrofluorescein diacetate staining revealed a significant ROS generation up to 2.6 to 3.2-fold increase in the cells treated with 62.5 µg/mL Cu_2_O NPs compared to the untreated controls, demonstrating robust antibacterial activity. The results suggest that lipopeptide biosurfactant stabilized Cu_2_O NPs could have promising potential for biocompatible bactericidal and therapeutic applications.

## Introduction

The genesis and an alarming spread of “multi-drug-resistant (MDR) bacteria” has become a severe peril to public health all over the world compromising the effectiveness of antibiotics^1, 2^. The increasing frequency of antibiotic resistance in many bacterial pathogens with subsequent failure of antibiotic therapy, especially in intensive care unit patients, has led to hundreds of thousands of deaths annually^3^. The calamity of antibiotic resistance has been attributed to the overuse and misuse of these drugs, along with the pharmaceutical industry's lack of new drug development due to reduced economic incentives and challenging regulatory requirements^4^. Discovery of new antibiotics and chemical modification of the existing antimicrobial drugs are among the exceedingly sought-after strategies to address the challenge of bacterial resistance to antibacterial drugs. Appallingly, there is no guarantee that new antimicrobial drugs can cope with the rapid and frequent development of resistance of the microbial pathogen in a timely manner^5^.

In recent efforts to address this challenge, metallic and metallic oxide nanoparticles have emerged as significant and novel antimicrobial agents^5–9^. Nanoparticles exhibit fascinating mechanical, magnetic, electrical and optical properties as well as high adsorption and catalytic competencies compared to their bulk counterparts owing to their nanodimensions (1–100 nm range)^1, 10–12^. Intrinsic tendency of boosted release of metallic ions and close interaction of nanoparticles with bacterial membranes which are accountable for antibacterial activity of nanoparticles can be attributed to their high surface area to volume ratio^9^. A variety of antibiotic resistant infectious diseases have been treated both in vitro and in vivo animal models by numerous classes of nanoparticles and nanoscale antibiotic carriers^1^. Nanoparticles provide a way to address “common antibiotic resistance mechanisms such as regulation of permeability, multi-drug efflux pumps, antibiotic degradation and target site binding affinity mutations”^13^. Diverse simultaneous mechanisms of action of nanoparticles against bacteria would make it hardly possible for the microbes to develop resistance, as the bacterial cell would be required to make multiple simultaneous gene mutations to develop this resistance^2^.

Recently, metallic copper, cupric oxide (CuO) and cuprous oxide (Cu_2_O) nanoparticles are gaining mounting attention due to their widespread application in electronic, optical sensors, catalysts and therapeutic applications^14–16^. Several studies have reported antibacterial activity of copper oxide NPs against Gram-positive bacteria, such as *B. subtilis, S. aureus* and Gram-negative bacteria such as, *P. aeruginosa* and *E. coli*^12, 17–20^. Metallic copper as well as copper oxide nanoparticles have exhibited multitoxicity to a broad-spectrum of bacterial species, including some multi-drug resistant bacteria such as the “superbug” MRSA (methicillin-resistant *S. aureus*)^21^. The growing attention on copper oxide nanoparticles is prompted by their cheaper price and abundance compared to the noble and expensive metals like gold, silver and their competent potential application as microbial agents^18^. Besides other mechanisms, mechanism of action of copper oxide NPs against microbes includes generation of oxidative stress and tendency of copper nanoparticles to alternate between cupric, Cu(II) and cuprous, Cu(I) oxidation states, making it unique from other metal nanoparticles^18^. Especially, Cu_2_O nanoparticles are widely abundant and have been reported to show lower toxicity, good environmental acceptability and remarkable broad-spectrum antibacterial and anti-superbug activity against a range of bacteria through generation of reactive oxygen species (ROS) and release of copper ions^22–25^. Zhou et al.^25^ reported remarkable antibacterial activity of Cu_2_O NPs on the superbugs “*methicillin-resistant staphylococcus aureus* (MRSA)” and “*vancomycin-resistant enterococcus* (VRE*)*” after being loaded on ZrP nanosheet matrix. Cuprous (Cu(I)) ions from Cu_2_O, have been shown to be considerably more toxic than cupric (Cu(II)) ions, due to their higher thiophilicity and cytoplasmic membrane permeability^26^.

However, the major limitation of metallic copper oxide particles in the nano-size range is lack of sufficient stability of their dispersions due to their strong tendency to aggregate and form larger clusters to reduce the energy associated with their high surface area^22, 25, 27^. The cluster formation is followed by rapid sedimentation leading to loss of reactivity and bactericidal applications in which a nanometric size is required^27^. Hence, several types of industrially produced chemical surfactants like “sodium dodecylbenzene sulfonate (SDBS), cetyltrimethylammonium bromide (CTAB), sodium bis(2-ethylhexyl) sulfosuccinate (AOT)” have been used as stabilizers to prevent aggregation, generate nanoparticles with uniform size distribution and improve long-term antimicrobial performance^28, 29^. Nonetheless, despite their widespread application they have limitation due to their petrochemical origin, thus the green alternatives will have indispensable option, as they would be biocompatible for various therapeutic and biomedical applications. Biosurfactants are natural surfactants synthesized by bacteria, fungi, animals and plants, offering several advantages compared to their chemical counterparts besides biocompatibility such as biodegradability, efficacy under extreme pH, temperature and salinity^30, 31^. Lipopeptides are structurally diverse group of biosurfactants predominantly produced by bacteria of genera *Streptomyces*, *Bacillus and Pseudomonas*^32, 33^.

In the current study, cuprous oxide nanoparticles (Cu_2_O NPs) are produced by chemical reduction of copper sulphate salts in water-in-oil microemulsion solution using NaBH_4_ as a reductant. Water-in-oil (w/o) microemulsions also called reverse micelles are water pools stabilized by surfactants, dispersed in oil phase, that serves as nanoreactors in which nanoparticles form and serve in controlling the size of nanoparticles^34, 35^. The water pools being stabilized by the lipopeptidal surfactant act both as nanoreactors for the process reaction and prevent particle aggregations as the surfactants get adsorbed on the particle surfaces when the particle size approaches the water pool, resulting in fine and uniform particle size distribution^35^. For the preparation of microemulsions, the lipopeptidal microbial surfactant previously synthesized in our laboratory was used. Scanning electron microscopy (SEM), Transmission electron microscopy (TEM), energy dispersive x-ray spectrum (EDX), X-ray diffraction (XRD) and UV–Vis analysis of the as-synthesized cuprous oxide nanoparticles was conducted, and antibacterial activity of the nanoparticles was assessed against *P. aeruginosa* CB1 and *B. subtilis* CN2 model Gram-negative and Gram-positive bacterial strains respectively. *Bacillus* spp. are increasingly utilized as non-hazardous biological food supplements and are used for large scale industrial production of enzymes and proteins^36^. Hence, despite limited scientific data of multidrug resistant *Bacillus subtilis* strains, there is an increasing concern over the transfer of antibiotic resistance genes, as *Bacillus *spp*.* in a number of commercially available probiotic products have shown to be resistant to several antibiotics^36, 37^. Hence, we have chosen the spore forming *Bacillus subtilis* strain as a model Gram-positive strain. The Gram-negative *Pseudomonas aeruginosa* strain has been recognized as an opportunistic pathogen, with inherent, acquired and adaptive resistance mechanisms to multiple classes of antibiotics^38, 39^.

## Results and discussion

### Characterization of copper oxide nanoparticles (Cu_2_O NPs)

Copper oxide nanoparticles were synthesized from copper sulphate pentahydrate (CuSO_4_·5H_2_O) metal precursor using NaBH_4_ as a reducing agent and microbial surfactant identified as lipopeptidal as a stabilizing agent. Formation of copper nanoparticles is easily discernible from the changes in the colour of the solution. The colour of the mixed solution changed immediately, shifting from a colourless to a dark brown dispersion, suggesting production of copper nanoparticles.

**Figure 1:**
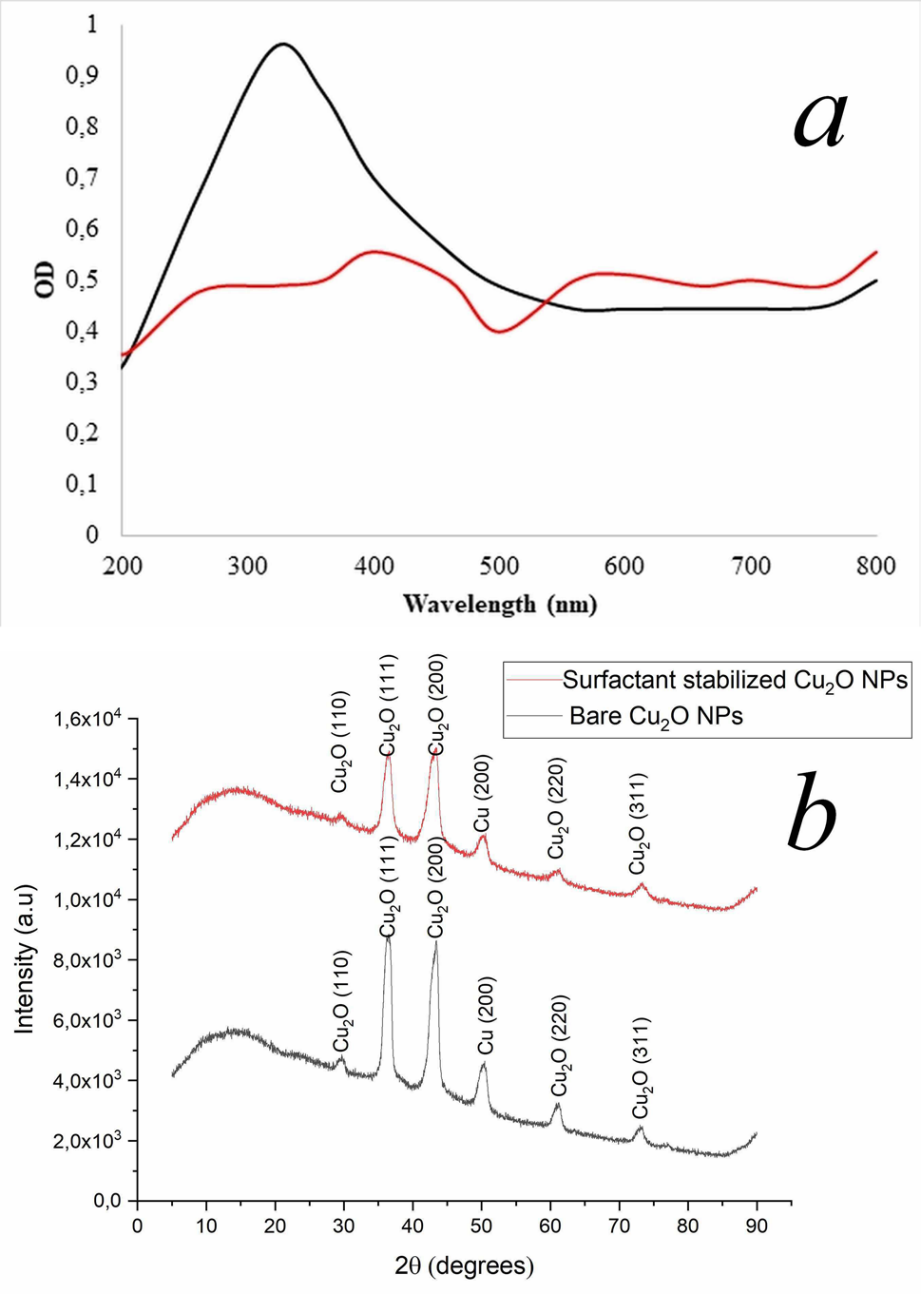
UV–Vis spectra of Cu_2_O NPs synthesised in the presence of lipopeptide microbial surfactant (**a**, black series) and in the absence of lipopeptide microbial surfactant (**a**, red series). Representative XRD patterns of the surfactant stabilized Cu_2_O NPs (**b**, red line) compared to the bare Cu_2_O NPs (**b**, black line), reflecting smaller size of the surfactant stabilized Cu_2_O NPs.

The absorption spectra of surfactant coated, and bare copper oxide nanoparticles are shown in Fig. 1a. The UV–Vis absorption spectra revealed an absorbance as portrayed in Fig. 1a (black line) from ~ 300 to ~ 350 nm suggesting formation of copper oxide nanoparticles^40, 41^. Albeit copper nanoparticles exhibit intense localized surface plasmon resonance in the visible region, the nanoparticles in the current study didn’t show any peak in the visible region, while distinct broad band was observed at ~ 320 for surfactant stabilized copper oxide nanoparticles. This could be attributed to the formation of cuprous oxide nanoparticles (Cu_2_O)^40, 42, 43^.

On the other hand, there was no characteristic surface plasmon resonance peak observed in the bare Cu_2_O NPs (Fig. 1a red line). This observation indicates that the bare Cu_2_O NPs undergo agglomeration to bigger particles^10^. In addition, “narrower and sharper peaks are indicative of more uniform particle size distribution of nanoparticles”^44^. The intensity of the surfactant stabilized nanoparticles peak remained sharper and in the same position for over two months, while there was no peak observed in the Cu_2_O NPs synthesized in the absence of the surfactant. In contrast to the Cu_2_O NPs synthesized in the absence of microbial surfactant, there had been hardly any precipitation and variation in the absorption properties of the surfactant stabilized nanoparticle suspensions stored in a sealed container, which lasted longer than two months, demonstrating their long-term colloidal stability.

X-ray diffraction (XRD) characterization was carried out to examine phase and purity of the as-synthesized products. Representative XRD patterns of the surfactant stabilized and bare Cu_2_O NPs (Fig. 1b) display a set of distinct diffraction peaks inferring the crystalline nature of the samples. The observed diffraction peaks of the samples at 2θ values of 29.40°, 36.5°, 61.40° and 73.10° could be indexed to (110), (111), (200), (220) and (111) planes of crystalline Cu_2_O, which is in agreement with the Cu_2_O powder peaks obtained from of the International Centre of Diffraction Data card (JCPDS file no. 05–0667). Besides, the XRD patterns show diffraction peak(s) corresponding to the Cu phase, however more than 95% by weight of the phase consists of Cu_2_O, indicating high purity of the as-synthesized cuprous oxide nanoparticles.

Intensities of XRD peaks reveal degree of crystallinity of the samples and peak broadening may indicate smaller crystallite size of the nanocrystalline material produced^45^. The high intensity XRD diffraction peaks of the Cu_2_O NPs reflect that the nanoparticles formed are highly crystalline and the broader diffraction peaks of surfactant stabilized Cu_2_O NPs (Fig. 1b, black line) compared to the bare Cu_2_O NPs (Fig. 1b, red line) reflect smaller size of the surfactant stabilized Cu_2_O NPs. Average crystallite size of the samples was calculated from XRD peak width based on Debye–Scherrer equation^45^.

1 $${\text{D}} = {\text{k}}\lambda /{\beta}{\cos }\theta$$

where D is the crystallite size, *k* is a constant (= 0.94 assuming that the particles are spherical), λ is wavelength of X-ray (0.1541 nm), β is full width at half maximum (FWHM) and θ is the diffraction angle. The crystallite sizes of the Cu_2_O NPs synthesized in the presence and absence of the biosurfactant were estimated to be 22.6 ± 4 nm and 67.5 ± 6 nm respectively.

**Figure 2:**
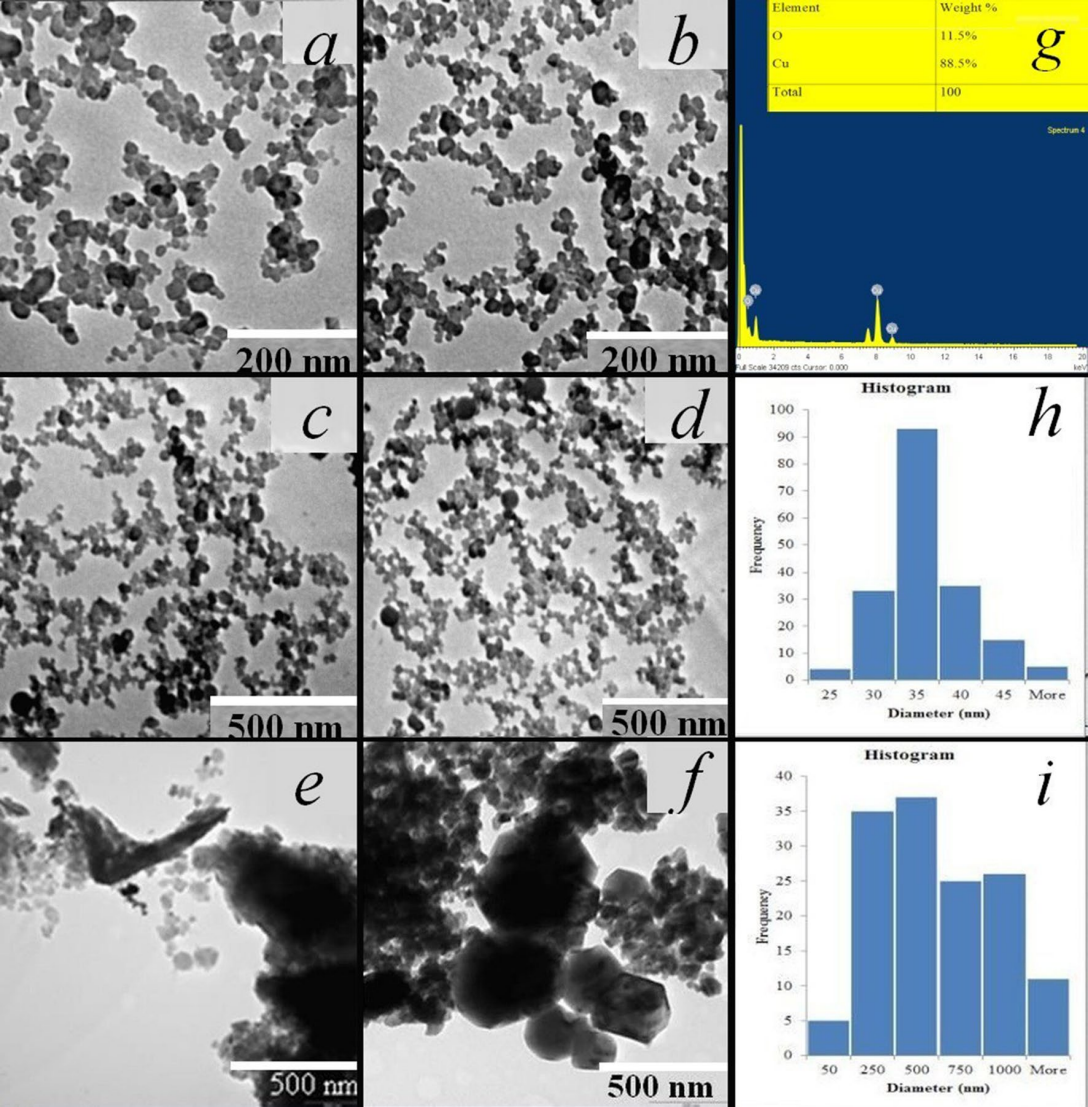
Representative TEM images of (**a**, **c**), Cu_2_O NPs at 1 g/L, (**b**, **d**), Cu_2_O NPs at 2 g/L lipopeptide biosurfactant additives at different magnifications; (**e**, **f**) Cu_2_O NPs with no lipopeptide biosurfactant additive, (**g**) EDX of the synthesised Cu_2_O NPs with an inset chart displaying percentage elemental composition of copper and oxygen. (**h**, **i**) Particle size distribution histograms of Cu_2_O NPs synthesised in the presence of 2 g/L lipopeptide biosurfactant additive and in its absence respectively.

In an experiment conducted to explore the effect of the microbial surfactant concentration on nanoparticle size and stability, the concentrations of the microbial surfactant at 0, 1 g/L and 2 g/L were evaluated, while keeping the concentration of metal precursor salt constant. The size and morphology of the as-synthesized Cu_2_O NPs were studied by scanning electron microscopy (SEM) and transmission electron microscopy (TEM) techniques. Figures 2a–f, h, and i show typical TEM images of Cu_2_O NPs synthesised in the presence of different concentrations of the microbial surfactant taken at different magnifications with their respective particle size distribution histograms. The elemental composition of the as-synthesised nanoparticles was confirmed through TEM equipped Energy dispersive X-ray spectroscopy (EDX) (Fig. 2g). As shown in Fig. 2g, EDX compositional analysis displayed spectra of elemental copper and oxygen, indicating oxidation of copper. The weight compositions of copper (Cu) and oxygen (O) were 88.50% and 11.50% by weight, presenting a stoichiometric ratio of Cu to O of 1.97:1, which is close to 2:1, displaying that the obtained products are cuprous oxide (Cu_2_O) particles. This result is comparable with that reported by Kooti and Matouri^46^.

TEM analysis demonstrated that the presence of the microbial surfactant played an important role in controlling the size and size distribution of Cu_2_O NPs. As revealed in Fig. 2e and f, when the synthesis of Cu_2_O NPs was carried out in the absence of the microbial surfactant lipopeptide, aggregated Cu_2_O particles with irregular shapes of mean microscale particle size ranging from ~ 250 to ~ 550 nm predominantly with a broad, multimodal particle size distribution were observed. Once the microbial surfactant at concentrations of 1 g/L and 2 g/L was added to the system, predominantly spherical nanoparticles were obtained with mean particle sizes of 35.5 ± 2 nm and 30.3 ± 2 nm respectively (Fig. 2a–d), suggesting that smaller nanoparticles were obtained at higher concentration of the surfactant administered. It can be observed that the particle sizes of Cu_2_O NPs determined from TEM are larger than the crystallite sizes of the Cu_2_O NPs determined from XRD peaks, the best explanation for this phenomenon is that a single particle may be comprised of several crystalline domains (crystallites).

In comparison with the bare Cu_2_O NPs, addition of surfactant at 1 g/L and 2 g/L exhibited a higher degree of nanoparticle uniformity and colloidal dispersion. This indicates that the size and size distribution of surfactant stabilized Cu_2_O NPs are dramatically reduced compared to the bare Cu_2_O NPs. Several parameters like surfactant structure and concentration, oil phase volumetric fraction and presence of co-stabilizers have impacts on colloidal stability, particles size and size distribution of nanoparticles synthesized in reverse micelles^47^. Water to surfactant ratio, ω_*o*_, which varies linearly with reverse micelle size is the most comprehensively exploited parameter in controlling particles size of nanoparticles obtained in the water-in-oil  microemulsion method^47^. Micelles in microemulsion systems, considered as nanoreactors, frequently collide via Brownian motion and exchange contents through formation of transient dimers, inside which the reactants get in to contact offering a favourable environment for controlled nucleation and growth. Furthermore, after the reaction in the soft templates or nanoreactors the steric stabilization provided by the surfactant avoids aggregation of the nanoparticles synthesized^48^. The significant effect of the microbial surfactant in limiting the nanoparticle size could be attributed to the surfactants limiting the micellar and subsequent nanoparticles’ final sizes or acting as an agent to increase the number of nuclei formed^49^. Thus, provided that that the rate of addition of copper precursor remains constant while adding more surfactants, would result in decreasing size of nanoparticles due to the larger number of nuclei.

**Figure 3:**
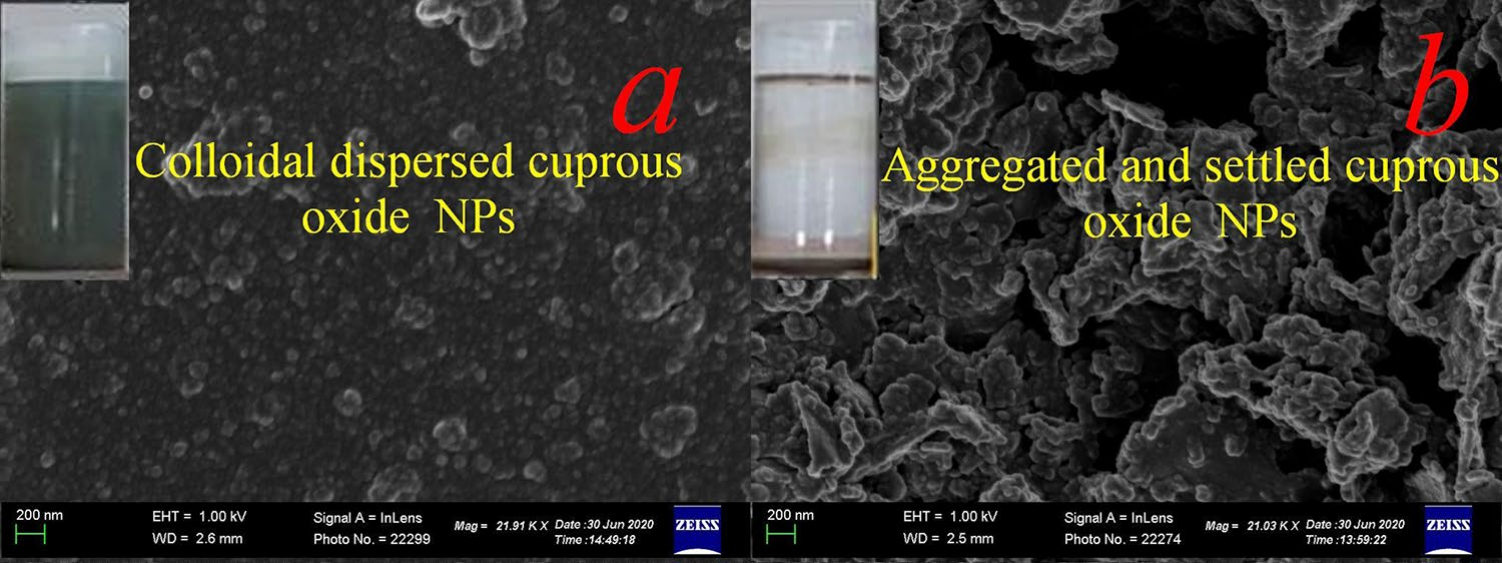
Representative SEM images of Cu_2_O NPs prepared (**a**) in the presence of 2 g/L lipopeptide surfactant, (**b**) in the absence of the lipopeptide microbial surfactant, displaying larger aggregated particles. Scale bar: 200 nm. The vials in the insets show colloidal stability of the resultant products after standing undisturbed for two weeks.

SEM analysis of the copper nanoparticles obtained in the presence and absence of the microbial surfactant provided more insight into the morphology of the Cu_2_O NPs (Fig. 3). As shown in Fig. 3a, the surfactant coated Cu_2_O NPs samples were uniform and well dispersed despite the different starting concentrations of surfactant in the medium. When the synthesis of the nanoparticles was performed without the lipopeptidal surfactant, poly-dispersed and cluster of aggregated nanoparticles with irregular shapes were obtained (Fig. 3b). It has been observed that, surfactant stabilization at the monitored dosages resulted in a significant reduction in the average size of the Cu_2_O NPs, indicating the important role the surfactant played in the formation process of Cu_2_O NPs confirming TEM results.

Closer observation of Fig. 3b reveals that the Cu_2_O NPs synthesized in the absence of the microbial surfactant have aggregated to form larger nanoclusters. The Cu_2_O NPs dispersed in aqueous solution could be preserved for only few hours as all the nanoparticles fell on the bottom due to the presence of larger aggregated Cu_2_O nanoclusters as portrayed in the insets of Fig. 3b. On the contrary, monodispersed surfactant coated Cu_2_O NPs remained dispersed in the aqueous medium for more than two months, as depicted in the insets of Fig. 3a. To achieve the maximum colloidal stability of the nanoparticles and prevent aggregation, long-range repulsion between the particles may be provided by electrostatic and steric stabilization mechanisms^50^. Steric stabilization results from steric barriers generated by surfactant or polymeric material adsorbates that surround the nanoparticles and prevent aggregation. Electrostatic stabilization is provided through formation of electrical double layers generated from ions adsorbed on the surface of nanoparticles which would result in coulombic repulsions between particles that would prevent agglomeration if it is sufficiently high^51^.

### Antibacterial activity of the Cu_2_O NPs

#### Evaluation of antimicrobial effect by minimum inhibitory concentration (MIC)

**Figure 4:**
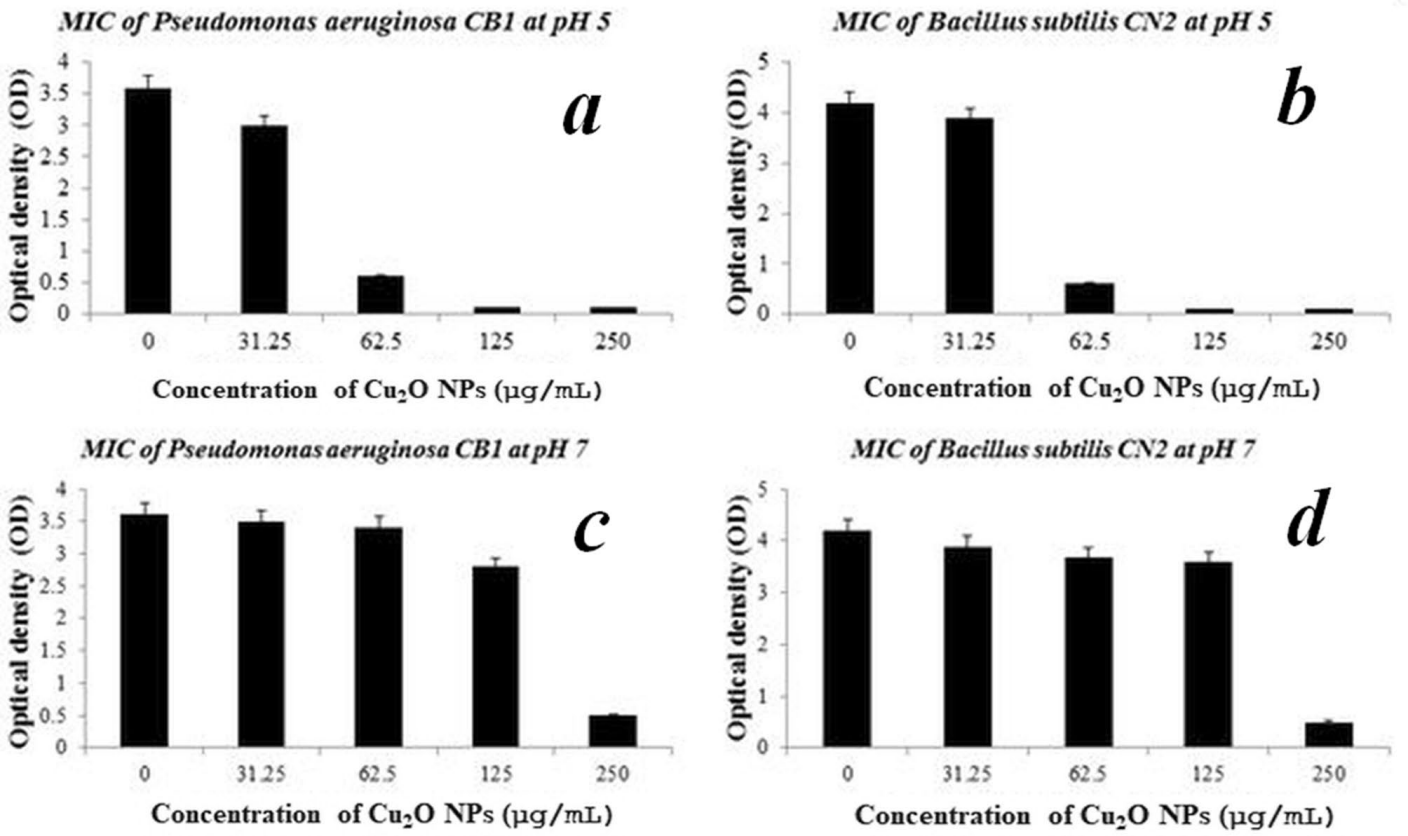
MIC of Cu_2_O NPs against *P. aeruginosa* CB1 and *B. subtilis* CN2 at different pHs evaluated by measuring optical density at 600 nm (OD_600_) after incubation at 37 °C, 180 rpm for 24 h. Data are presented as mean ± SD of three independent experiments, each performed in triplicates. Variation between sample means are analysed by *ANOVA.*

Antimicrobial activity of the smaller Cu_2_O NPs synthesised at 2 g/L lipopeptide was evaluated against Gram-negative *Pseudomonas aeruginosa* CB1 and Gram-positive *Bacillus subtilis* CN2 bacterial strains as the minimum inhibitory concentration (MIC) at varying pH values, as summarized in Fig. 4. The MIC value of Cu_2_O NPs against both the Gram-negative *Pseudomonas aeruginosa* CB1 and Gram-positive *Bacillus subtilis* CN2 strains was found at 66.5 µg/mL at pH5 (Fig. 4a,b). On the other hand, at pH 7 the copper nanoparticles displayed significantly lower antibacterial activity at 250 µg/mL (Fig. 4c,d), suggesting that Cu_2_O NPs were more toxic and effective against both the microbial strains in a slightly acidic condition. However, no antibacterial activity of the copper nanoparticles was observed at pH values higher than 7, suggesting the decrease in antibacterial activity of the cuprous oxide nanoparticles at higher pH values.

In contrast, bare nanoparticles did not show significant antibacterial effect at any of the pH values monitored compared to the surfactant stabilized and monodispersed nanoparticles (data not shown). It is observed that the antibacterial activity of the nanoparticles is dependent on pH, size and colloidal dispersion of the nanoparticles. Thus, the smaller size and colloidal stability of the Cu_2_O NPs provided by the microbial surfactants has a remarkable effect on the antibacterial activity of the nanoparticles. In a similar study by Hsueh et al.^19^, CuO NPs exhibited significant toxicity at pH 5 against USA300, and ATCC6538, Newman, and SA113 *Staphylococcus aureus* strains and did not show significant antibacterial activity at pH 7 and 6.

### The agar-well diffusion test

The Cu_2_O NPs demonstrated remarkable antibacterial activity against both the Gram-positive *B. subtilis* CN2 and Gram-negative *P. aeruginosa* CB1 strains in the agar-well diffusion test with 100µL volumes per well (Table 1). The extent of inhibitory effect on bacterial growth was observed to be dose and colloidal stability dependent.

**Table 1:** Inhibition zone of Cu_2_O NPs against the Gram-positive *B. subtilis* CN2 and the Gram-negative bacteria *P. aeruginosa* CB1 strains.

Concentration	Zone of inhibition (mm)			
	*P. aeruginosa* CB1		*B. subtilis* CN2	
	Stabilized Cu_2_O NPs	Bare Cu_2_O NPs	Stabilized Cu_2_O NPs	Bare Cu_2_O NPs
1 mg/mL	14.6 ± 1.0	4.5 ± 0.7	16.1 ± 1.0	5.30 ± 0.5
2 mg/mL	26.5 ± 2.0	9.7 ± 0.9	28.6 ± 1.2	9.8 ± 1.1

The results are represented as mean ± SD of three independent experiments, each done in triplicates.

Supplementary Fig. S1a and b illustrate the result of agar well diffusion tests performed with 1 mg/mL and 2 mg/mL Cu_2_O NPs concentrations. The smaller sized surfactant stabilized Cu_2_O NPs showed a significant dose-dependent growth inhibition against both *B. subtilis* CN2 and *P. aeruginosa* CB1 compared to the bare Cu_2_O NPs (Table 1). The bare Cu_2_O NPs demonstrated lower zones of inhibition for both *P. aeruginosa* CB1 and *B. subtilis* CN2, which were approximately one-third the zones of inhibition observed for the surfactant stabilized NPs, reflecting the higher diffusion of the surfactant stabilized smaller Cu_2_O NPs in the solid agar.

### Cell viability assay

**Figure 5:**
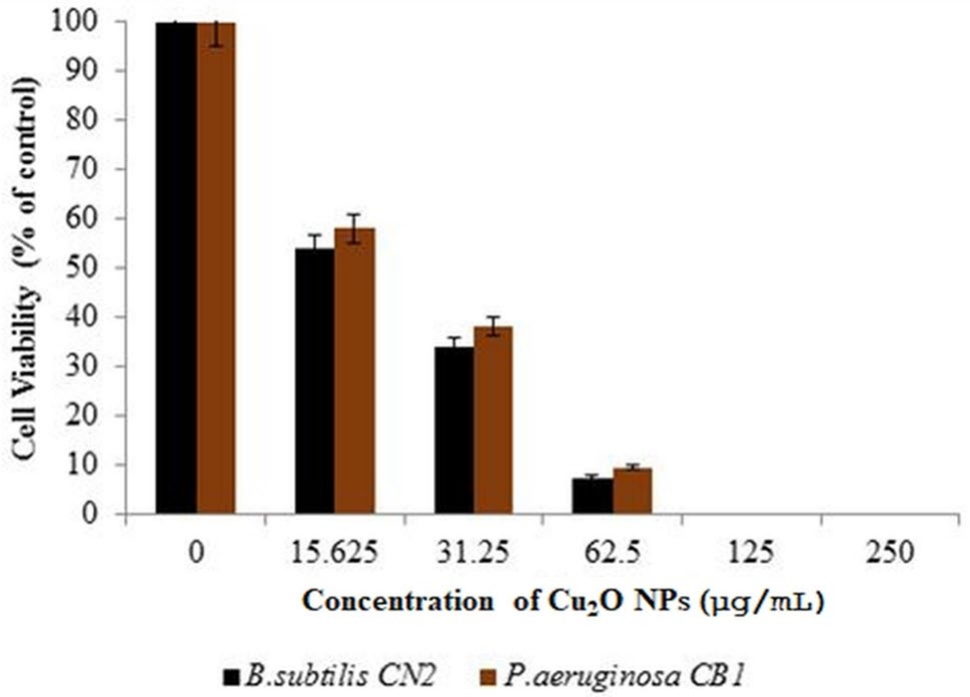
Cell viability percentages of Gram-positive *B. subtilis* CN2 and Gram-negative *P. aeruginosa* CB1 cells against various concentrations of Cu_2_O NPs after 24 h of exposure; results are expressed as percentages of control cells. Values are mean ± SD of three independent experiments, each performed in triplicates and considered statistically significant when *p* < 0.05.

An MTT assay was performed to measure cytotoxicity of Cu_2_O NPs on the *P. aeruginosa* CB1 and *B. subtilis* CN2 cells at 0, 15.625, 31.25, 62.5, 125, and 250 μg/mL concentrations after 24 h of incubation. The Cu_2_O NPs exhibited high degree of cytotoxicity at 62.5 µg/mL and higher concentrations, which are statistically significant compared to untreated control cells (*p* <  0.05), while 32.25 and 15.625 μg/mL concentrations showed no significant differences with the control group (*p* > 0.05). As indicated in Fig. 5, the cytotoxicity of the nanoparticles is dependent on their dosage. Among the two strains, slightly higher cytotoxicity was observed on *B. subtilis cells*. The half maximal inhibition concentration (IC_50_) value of Cu_2_O NPs required for 50% growth inhibition, is determined to be 21.21 μg/L and 18.65 μg/mL for *P. aeruginosa* and *B. subtilis* respectively after 24 h exposure. Cu_2_O NPs exhibited a slightly lower toxicity (higher IC_50_) towards the Gram negative *P. aeruginosa* cells.

### Mechanism of antimicrobial activity

#### TEM and SEM ultrastructure analysis

To elucidate the principal antibacterial mechanism of action of the Cu_2_O NPs we examined the effect of the nanoparticles on the bacterial cell morphology and changes in the cellular ultrastructure.

**Figure 6:**
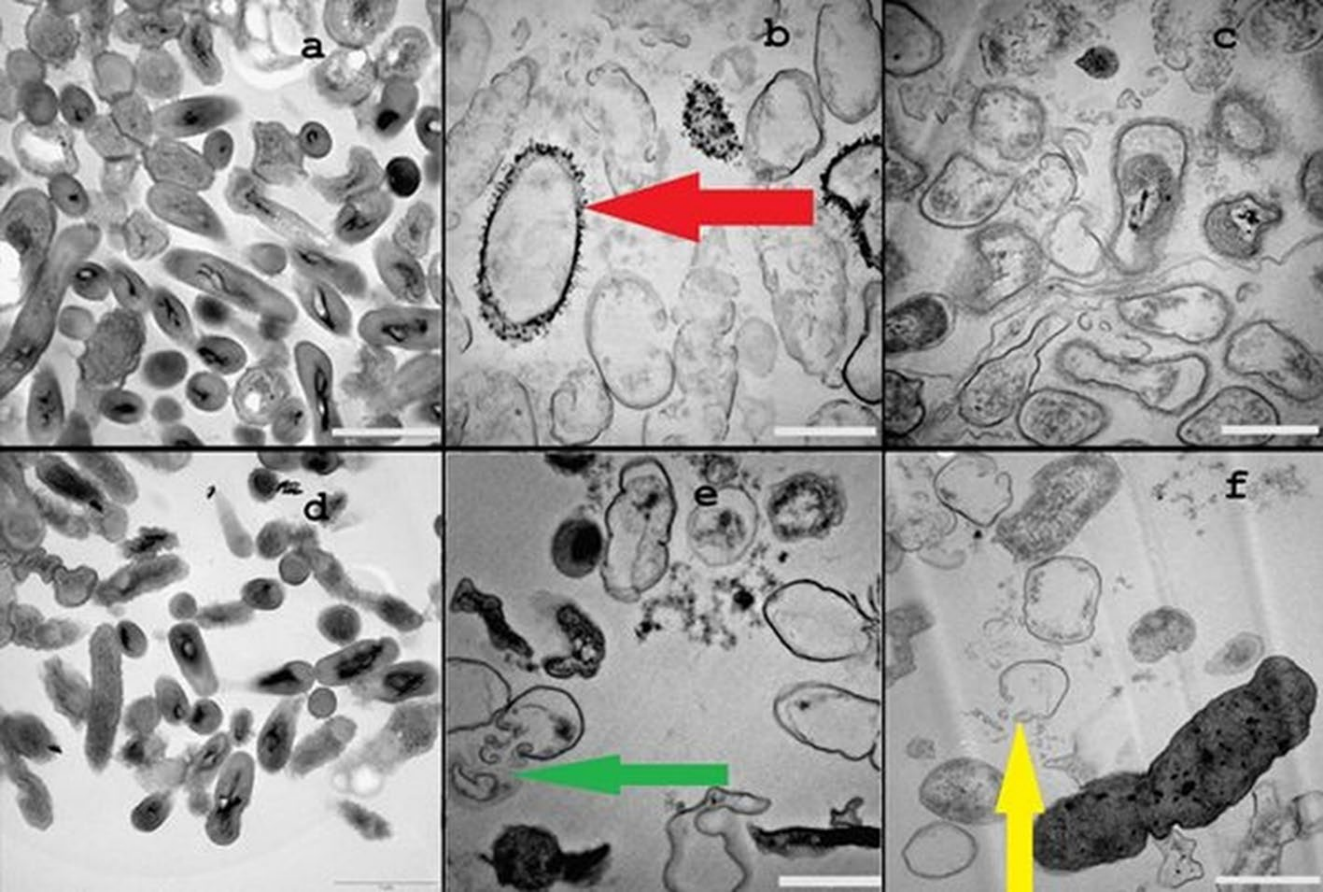
Representative TEM micrographs of untreated *B. subtilis* CN2 cells (**a**), showing intact and high electron density morphology, 100 µg/mL (**b**), 125 µg/mL (**c**) dosage of Cu_2_O NPs treated cells indicating cytoplasmic injury with disintegrated outer membrane (**f**, yellow arrow). *P. aeruginosa* CB1 cells treated with 100 µg/mL (**e**), 125 µg/mL (**f**) dosage of Cu_2_O NPs and untreated control (**d**). Considerable size of adhered nanoparticles was observed (**b**, red arrow) attached to the surface of the cells of the bacteria, and disrupted cell wall and membrane leakage was observed (**e**, green arrow). Scale bar is 1 µm.

The surface ultrastructure of the microbial cells was examined using TEM to visualize the subsequent morphological changes on bacteria cells following exposure to the Cu_2_O NPs and compared with their untreated controls (Fig. 6a–f). Figure 6a and d presents TEM micrographs of untreated *B. subtilis* CN2 and *P. aeruginosa* CB1 control cells respectively with intact cell membrane and cell wall with distinct morphology. It can be observed that the cells were short rod shaped, had a uniform electron density, suggesting that the cells were in a normal condition. After exposure to different concentrations of the Cu_2_O NPs the bacterial cells showed significant morphological changes to the shape and integrity (Fig. 6b–c, e–f). TEM images of treated cells show noticeable disruptions in membrane integrity with lots of cell debris due to cell rupture forming aggregated mass. Nanoparticles adherence to bacterial body with associated detachment of bacterial cell wall from the outer membrane were observed (Fig. 6e, green arrow). The TEM images displayed prevalent low-density region in the Cu_2_O NPs treated cells, suggesting severe cytoplasmic damage (Fig. 6f, yellow arrow). Cell wall and cytoplasmic membrane rupture with the concomitant outflow of internal cellular contents and collapse of cells was clearly observed (Fig. 6e–f). Figure 6d–f shows the ultrastructure of *B. subtilis* CN2 and *P. aeruginosa* CB1 to be remarkably changed after exposure to copper nanoparticles. Several Cu_2_O NPs were observed attached on the surface of *B. subtilis* CN2, displaying low density region due to permeability of the cell wall and leakage of cytoplasmic content (Fig. 6b, red arrow). A low-density region was observed throughout the Cu_2_O NPs treated cells, suggesting loss of integrity of membrane and leakage of cytoplasm (Fig. 6f, yellow arrow).

**Figure 7:**
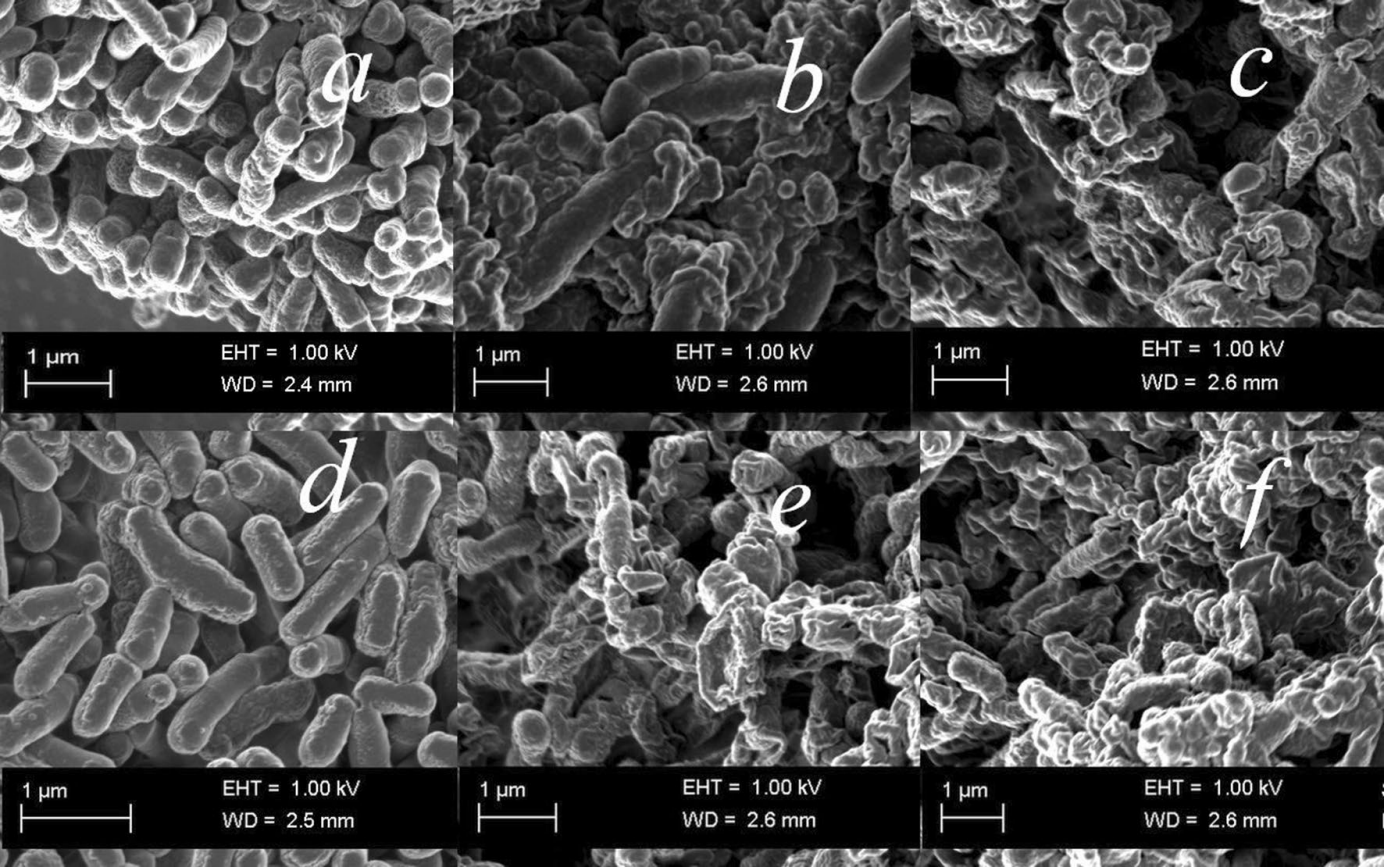
Representative SEM micrographs of untreated *B. subtilis* CN2 cells (**a**), showing intact and high electron density morphology, 100 µg/mL (**b**), 125 µg/mL (**c**) dosage Cu_2_O NPs treated cells indicating cytoplasmic injury with disintegrated outer membrane. *P. aeruginosa* CB1 untreated cells (**d**), treated with 100 µg/mL (**e**) and 125 µg/mL (**f**) dosage of Cu_2_O NPs. Scale bar is 1 µm.

To verify the results of TEM, scanning electron microscopy (SEM) observation was carried out to visualize the distinct morphological changes on the bacterial membranes treated with different concentrations of surfactant stabilized Cu_2_O NPs (Fig. 7). The results showed clear differences in the membrane morphology of the untreated and Cu_2_O NPs treated *B. subtilis* CN2 and *P. aeruginosa* CB1 cells. The untreated bacterial membranes remained intact, plump and evenly shaped (Fig. 7a and d). While the predominant cells of both Gram-negative and Gram-positive cells treated with the copper nanoparticles showed cell wall and membrane disruptions, withered morphology with leakage of intracellular substances and complete cell lysis consistent with TEM observation.

### Measurement of intracellular ROS

To elucidate the other proposed mechanism of toxicity of the Cu_2_O NPs species, an assay measuring cellular ROS generation have been employed on both *B. subtilis* CN2 and *P. aeruginosa* CB1 strains. Induction of cellular oxidative stress due to ROS formation has been attributed to be one of the principal bactericidal mechanisms of action of metal nanoparticles^10, 41, 52, 53^. To examine if the toxicity observed in the Cu_2_O NPs studied is related to the ROS induced oxidative stress, the level of cellular oxidative stress triggered by Cu_2_O NPs at increasing dosages (0, 31.25, 62.5, 125 µg/mL) was measured by flow cytometer (FACS) using H_2_DCFDA staining method, which fluoresces in response to ROS inside the cells to fluorescent DCF^54^. Thus, the magnitude of fluorescent intensity is proportional to the amount of ROS generated inside cells and the fluorescent signal was collected in the FL1 channel of FACS.

**Figure 8:**
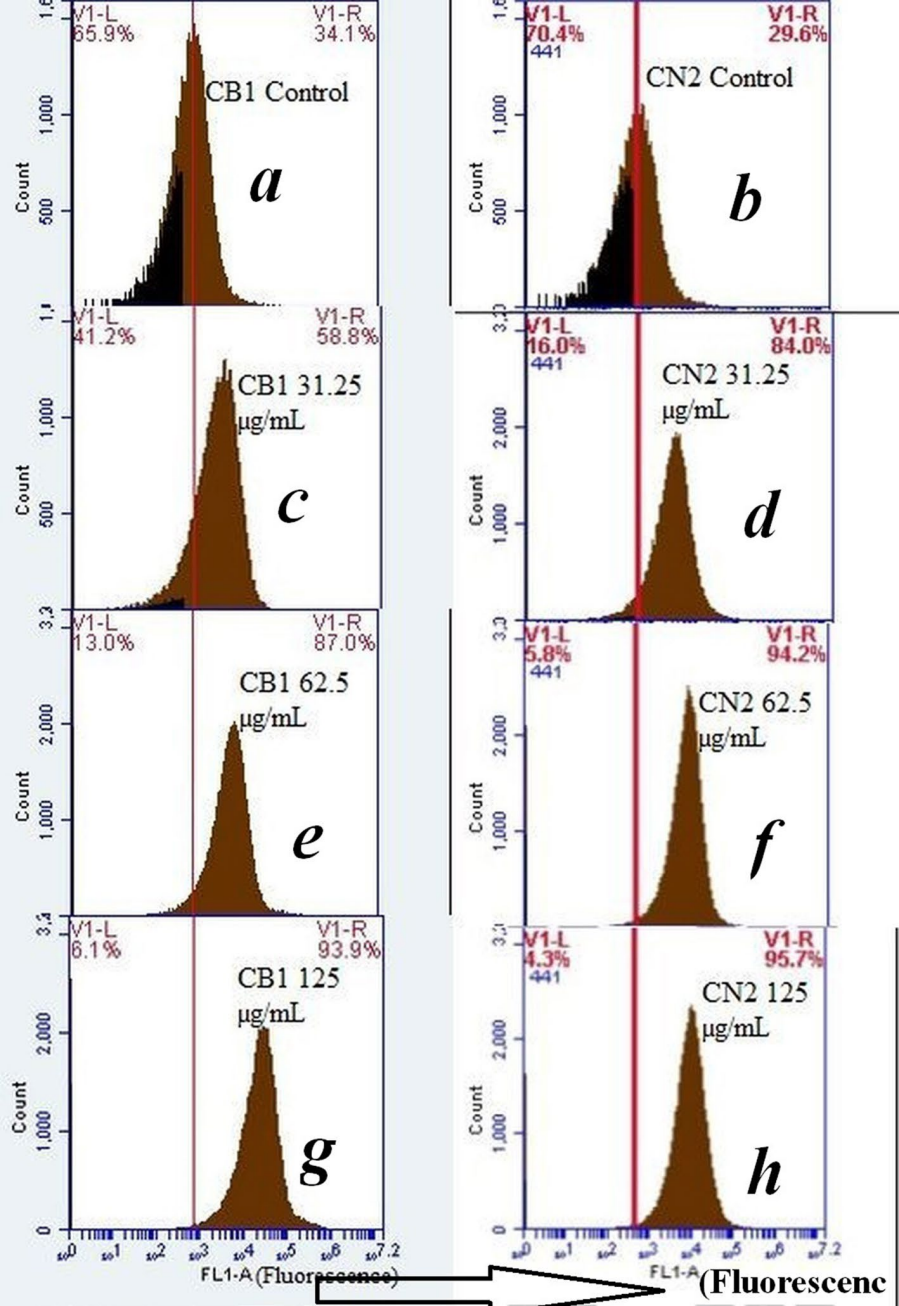
Oxidative stress response at various doses of Cu_2_O NPs on the Gram-negative *P. aeruginosa* CB1 (left column) and Gram-positive *B. subtilis* CN2 (right column) bacterial cells. (**a**, **b**) Control cells; (**c**, **d**) cells treated with 31.25 µg/mL; (**e**, **f**) cells treated with 62.5 µg/mL; (**g**, **h**) cells treated with 125 µg/mL for 24 h. After treatment of cells with designated concentrations of Cu_2_O NPs for 24 h, intracellular ROS generation was quantified by oxidation of cell permeable dye 2,7-dichlorodihydrofluorescein diacetate (DCFDA) staining using flow cytometer. The DCF fluorescence is proportional to the ROS generated. The FL1-A corresponds to the green emission of the DCF. The V1-L and V1-R markers correspond to ROS negative and positive cells respectively. Scale Bar: 5 µm.

The results of the flow cytometer (FACS) based ROS measurement demonstrated an increasing dose dependent build-up of ROS in both the Gram-negative *P. aeruginosa* CB1 (Fig. 8a, c, e, g) and Gram-positive *B. subtilis* CN2 (Fig. 8b, d, f, h) strains at 0, 31.25, 62.5 and 125 µg/mL Cu_2_O NPs dosages respectively. Treatment of the cells with 62.5 µg/mL Cu_2_O NPs dosage, which is the MIC value, exhibited 94.2% and 87% ROS positive cells on the Gram-positive and Gram-negative cells respectively compared to 29.6% and 34.1% ROS positive cells in their respective untreated controls. The results demonstrated significant 3.2-fold and 2.6-fold increase in cellular ROS level in the Gram-positive *B. subtilis* CN2 and Gram-negative *P. aeruginosa* CB1 cells respectively compared to the level in untreated controls (*p* < 0.05), due to exhaustion of antioxidant defence system. The results displayed a slight resistance to ROS on Gram-negative cells compared to the Gram-positive cells, which is in line with the cell viability assay. The higher sensitivity of Gram-positive bacteria to Cu_2_O NPs is further confirmed in a study carried out at a set of diverse dosages as well (Supplementary Fig. S2). However, there is no statistically significant change between the ROS values of the strains (*p* > 0.05), demonstrating broad-spectrum activity of the nanoparticles in generating ROS on the strains.

**Figure 9:**
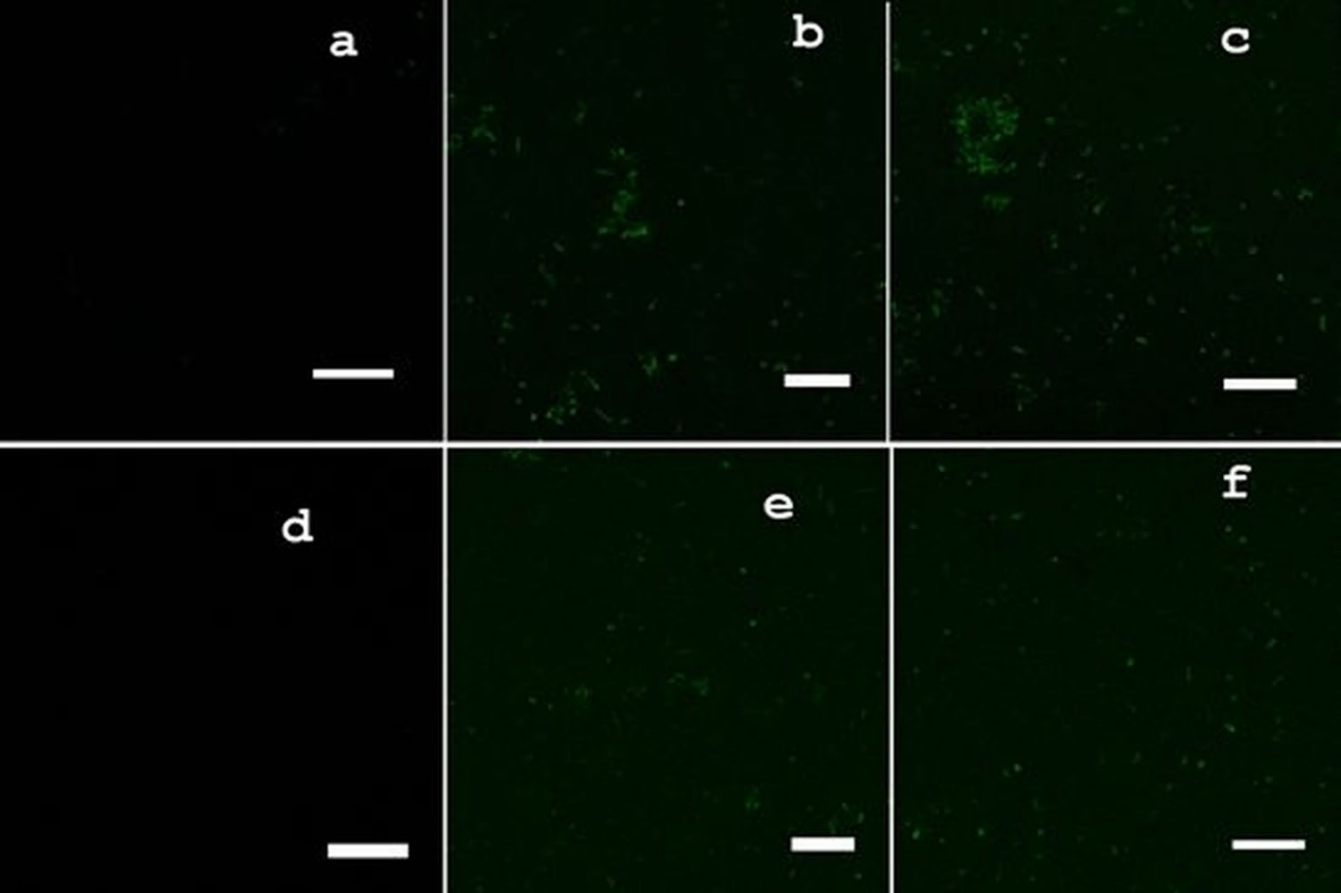
Confocal microscopy of green fluorescence images of ROS in Cu_2_O NPs treated, and untreated control cells measured by 2,7-dichlorofluorescin diacetate (DCFH-DA) fluorescence-based assay. Cells were treated with 0, 62.5 and 125 μg/mL of surfactant stabilized Cu_2_O NPs for 24 h. (**a**–**c**) green fluorescence images of 0, 62.5 and 125 μg/mL dose Cu_2_O NPs exposed Gram-positive *B. subtilis* CN2 cells*.* (**d**–**f**) green fluorescence images of 0, 62.5 and 125 μg/mL dose Cu_2_O NPs exposed Gram-negative *P. aeruginosa* CB1 cells*.* Untreated cells were used as a negative control (**a**, **d**). Scale Bar: 5 µm.

Confocal microscopy assisted visualization of the green fluorescence using DCFH-DA probe confirmed significant ROS generation in Cu_2_O NPs treated cells (Fig. 9b, c, e and f) compared to the untreated controls (Fig. 9a and d). The confocal microscopy green fluorescent images demonstrated that exposure of cells to Cu_2_O NPs induced dose dependent ROS generation proportional to the fluorescent intensity. Both the Gram-positive *B. subtilis* CN2 (Fig. 9b and c) and Gram-negative *P. aeruginosa* CB1 (Fig. 9e and f) cells confocal micrographs exhibited enhanced ROS generation in comparison to the ROS generated in their respective untreated controls (Fig. 9a and d, respectively). Significant ROS generation occurred in both Gram-positive and Gram-negative strains demonstrating non-specificity and broad-spectrum oxidative stress induction potential of the copper nanoparticles. It can be observed that consistent with the results from the growth inhibition assay, the overall toxicity of the copper nanoparticles against both Gram-positive and Gram-negative bacteria is strongly correlated with cellular ROS accumulation (r^2^ ≅ 9.6).

### Dissolution and cellular uptake of Cu_2_O NPs

One possible explanation for substantial toxicity of Cu_2_O NPs has been attributed to the release of Cu^+^ ions from the Cu_2_O NPs or uptake of Cu_2_O NPs by the cells. To test this possibility, we conducted dissolution study and copper intracellular uptake assay.

**Figure 10:**
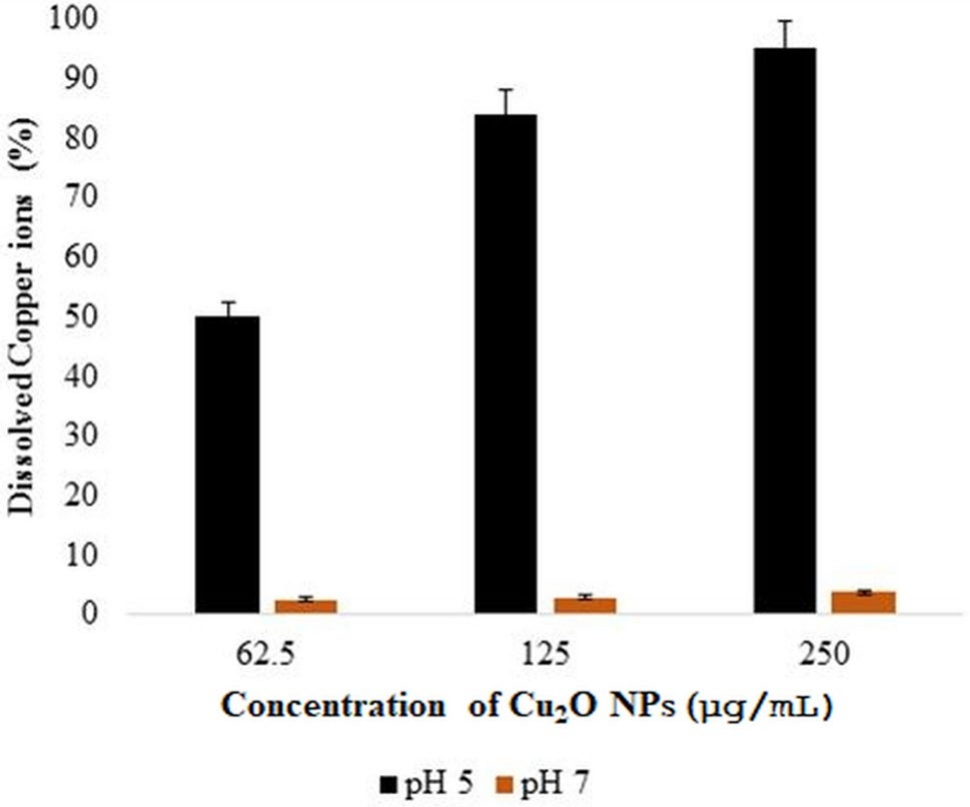
Percentage of dissolved copper ions released from various dosages of Cu_2_O NPs at different pH values after 24 h of exposure at 37 °C. Data are means ± standard deviations of three independent experiments each performed in triplicates. There was no statistically significant difference between the amount of Cu^1+^ dissolved at 62.5, 125, and 250 µg/mL treatments at the pHs administered.

The results of the study showed that dissolution of Cu^2^ ions from copper nanoparticles was significantly higher at pH 5 compared to pH 7 (*p* < 0.05), as depicted in Fig. 10. There was 50%, 84% and 90% dissolution of Cu^+^ ions at pH 5 compared to 2.4%, 2.8% and 3.5% dissolution of copper ions at pH 7 at 62.5, 125 and 250 µg/mL Cu_2_O NPs concentrations respectively. In a similar study Cai et al.^55^, reported that “less than 0.1% of the nano-Cu dissolved in 48 h in the freshwater media at a higher pH value of 8.2 compared to 98% dissolution of nano-Cu at a lower pH value of 6, demonstrating the importance of pH and media composition on CuNPs’ dissolution”. This suggests that the remarkably high sensitivity of the Gram-positive and Gram-negative bacteria to Cu_2_O NPs at pH 5 compared to pH 7 could be due to the elevated cuprous ions released at lower pH value. As depicted on Fig. 4a,b, in the minimum inhibitory concentration study the higher toxicity of Cu_2_O NPs at pH 5 (MIC values of 62.5 µg/mL at pH 5 compared to 250 µg/mL at pH 7 ) could be attributed to the significantly high amount of cuprous ions dissolved at pH 5 compared to pH 7(*p* < 0.05).

**Figure 11:**
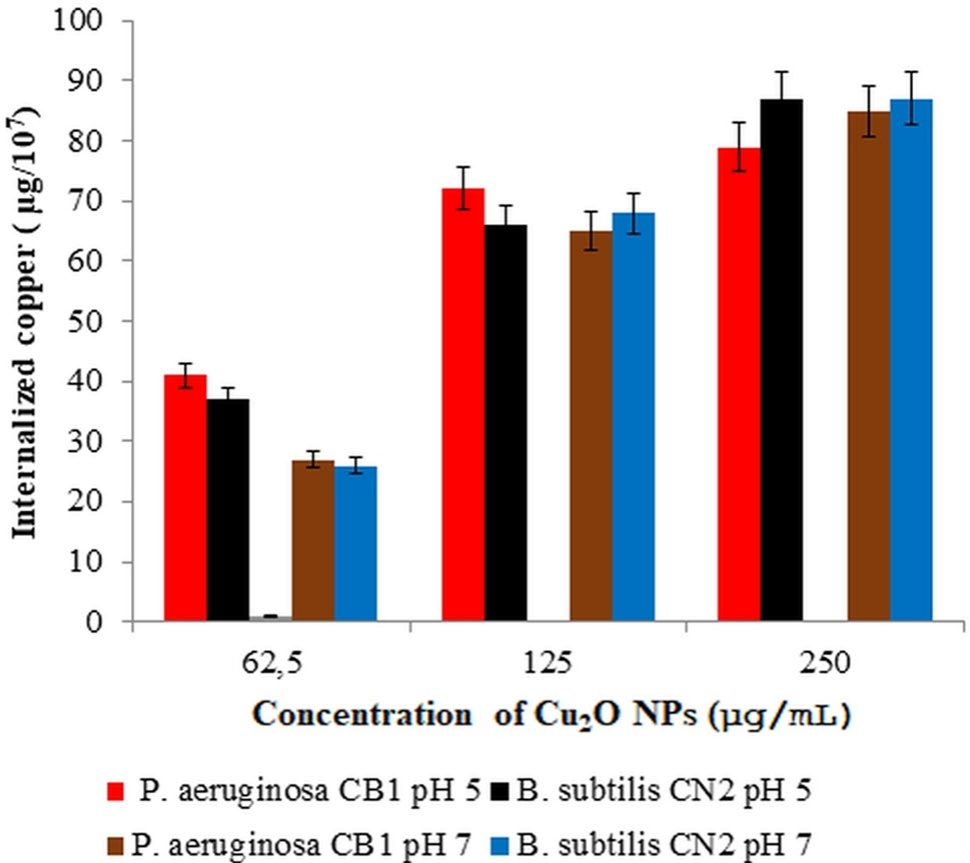
Cellular uptake of Cu_2_O NPs after 24 h of exposure of bacterial strains to various dosages of Cu_2_O NPs at 37 °C. Data are means ± standard deviations of three independent experiments each performed in triplicates. There was no statistically significant difference between the amount of copper internalized at 62.5, 125, and 250 µg/mL treatments at pH 5 and pH 7 in each of the strains (*p* > 0.05).

After 24 h of exposure dose dependent cellular uptake of copper was observed (Fig. 11). The intracellular concentration of Cu was 41, 76, 79 µg/10^7^ cells and 37, 66, 72 µg/10^7^ cells for *P. aeruginosa* CB1 and *B. subtilis* CN2 strains respectively at 62.5, 125, 250 µg/mL Cu_2_O NPs dosages at pH 5, and there was no significant difference between the strains in assimilation of copper (*p* > 0.05). Comparative amount of copper was internalized by both the Gram-positive and Gram-negative strains at pH 7 and there was no significant change in the amount of copper internalized at pH 5 and pH 7 at the Cu_2_O NPs dosages (*p* > 0.05). Our results are in line with previous studies that the internalization of nanoparticles was size- and concentration-dependent^17, 56^. Cellular uptake and related particle-related toxicity of internalized nanoparticles has been demonstrated as one mechanism of cytotoxicity of CuO NPs^10^. Cu_2_O NPs can enter the cells “through diffusion, endocytosis or the action of carrier proteins, and react with intracellular components, leading to the disintegration of cells and cell contents”^56^. After penetration of Cu_2_O NPs into the cells, the nanoparticles would interact with mitochondria, vacuoles, ribosomes internal structures and biomolecules like protein, lipid and DNA, which would lead to loss of cell viability^57^.

The results of the study revealed broad-spectrum antibacterial activity of Cu_2_O NPs that can inhibit the growth of both Gram-positive *B. subtilis* and Gram-negative *P. aeruginosa* strains at pH 5*.* The significant antibacterial activity of the surfactant coated, and monodispersed smaller nanoparticles observed at lower pH value can be attributed to both the nano size effect and enhanced dissolution of Cu^+^ ions at pH 5.

In contrast, bare nanoparticles did not show significant antibacterial effect at any of the pH values monitored. This is due to agglomeration and lack of dispersion of the Cu_2_O NPs, which is of paramount necessity for maximal contact between microbes and Cu_2_O NPs and effective antibacterial activity^9^. Thus, the small size and colloidal stability provided by the microbial surfactant has a remarkable effect on the antibacterial activity of the nanoparticles besides the pH influence. The higher antibacterial activity may be attributed to higher solubility of Cu^+^ ions from the smaller sized, monodispersed Cu_2_O NPs compared to the bare and aggregated larger nanoparticle synthesized in the absence of the microbial surfactant. The lower agglomeration and high colloidal stability of the surfactant stabilized Cu_2_O NPs provided greater surface area for their interaction with bacterial membranes and for boosted dissolution of copper ions leading to enhanced toxicity of NPs^1^.

Furthermore, the surfactant stabilized Cu_2_O NPs displayed a slightly higher antibacterial activity against *B. subtilis* than *P. aeruginosa.* The slight variation in antibacterial sensitivity between the strains might be attributed to the cell wall structure and composition. The complex peptide double layer of the Gram-negative bacteria that consists of a thin peptidoglycan layer adjacent to cytoplasmic membrane and an outer membrane, prompted slight reduction in antibacterial sensitivity as it avoids penetration of the Cu_2_O NPs. On the other hand, the Gam-positive bacteria *B. subtilis* composed of only thick peptidoglycan layer was more susceptible to intracellular transduction causing cell wall disruption^20^. Hence, despite slight variations in antibacterial sensitivity the Cu_2_O NPs exhibited a wide spectrum of antimicrobial activity against both Gram-negative and Gram-positive bacteria.

While the antibacterial activity of Cu_2_O NPs is appealing, applications of Cu_2_O NPs are often hampered by their aggregation and subsequent loss of antibacterial activity. The zone of inhibition antibacterial assay generally revealed that surfactant stabilized, and smaller nanoparticles demonstrated remarkable antibacterial activity. The higher antibacterial activity of the smaller surfactant stabilized Cu_2_O NPs might be attributed to the high surface area with corresponding larger percentage of atoms at the surface causing enhanced reactivity and bacterial proximity for increased amount of Cu^+^ released in cell surroundings. Hence, regardless of relatively thicker cell wall of Gram-positive bacteria, the effective antibacterial activity of surfactant stabilized Cu_2_O NPs observed might be ascribed to the Cu^+^ ions released^10, 17^. Kaweeteerawat et al.^17^ demonstrated that nano sized Cu particles showed an enhanced and different mechanism of antibacterial activity compared to their micronized and ionic analogues. The authors demonstrated that the copper nanoparticles were strongly bound to *E. coli* and perceived to produce a significant amount of ROS and cause exceedingly detrimental damage to DNA in vitro.

The degree of toxicity and antibacterial activity of the Cu_2_O NPs depends on the combination of several factors like aeration, pH, concentration of nanoparticles and concentration of microbes^1^. The high temperature, high aeration and colloidal dispersion avoid agglomeration and increase the toxicity. Colloidal stability and higher surface area of smaller nanoparticles provide higher rate of solubilization of copper ions and larger surface rea for interaction with bacterial body offering enhanced toxicity^1, 9^. Copper nanoparticles have higher solubility at lower pH, facilitating enhanced release of copper ions that attack microorganisms effectively^9^.

The current growth inhibition concentrations are lower than the values previously reported^17, 19^. Hsueh et al*.*^19^, demonstrated that CuO NPs showed excellent bactericidal activity against four different *Staphylococcus aureus* strains at a concentration as high as 20 mM (1600 µg/mL). On the other hand, inhibitory concentrations of copper–polyaniline (Cu–PANI) nanocomposite as low as 20 µg/mL have been reported to completely inhibit growth of E*. coli and S. aureus* strains^18^. Generally, the MIC values of copper nanoparticles differ based on strain employed, initial bacterial concentration, shape and size of the nanoparticle used, thus it will not be pertinent enough to compare values from different studies.

The results of the microbial growth inhibition study according to the agar well diffusion method showed concentration and size dependent zones of inhibition at both the Gram-negative and Gram-positive strains confirming MIC results. This demonstrated that surfactant stabilized Cu_2_O NPs, being smaller readily diffuse into the agar medium, allowing greater interaction between the Cu_2_O NPs and the pathogens, exhibiting better microbicidal property. The zone of inhibition values in the current study are comparatively higher than the zone of inhibition values of Cu_2_O NPs previously reported in similar studies^20, 30^.

Although the mechanisms of antibacterial action of nanoparticles have not yet been fully elucidated, metallic nanoparticles and their related ions induced reactive oxygen species (ROS) generation, causing cell damage due to oxidative stress; adhesion and dissolution of metallic nanoparticles on bacterial membrane with subsequent permeability, disruption of membrane functionality and dissipation of the protein motive force have been reported as the main mechanisms^41, 58^. The observed cellular toxicity and inhibitory effect of the Cu_2_O NPs may be attributed to the ions released into the media or particle related effect of the nanoparticles^17^. The Cu_2_O NPs interact favourably “with the negatively charged bacterial cell membrane by electrostatic attraction, covalent or Vander Waals forces causing an increase in membrane permeability and eventually rupture and leakage of intracellular components”^59^. Both the Gram-positive and Gram-negative bacteria have negatively charged cell membranes favouring electrostatic interaction with the copper ions and the Cu_2_O NPs. In Gram-positive bacteria the anionic polysaccharide teichoic acid is playing a major role in interacting with the Cu_2_O NPs and ions released while in Gram-negative bacteria lipopolysaccharides and proteins present in the outer membrane carry out electrostatic stabilization of the copper ions and nanoparticles^9, 60^. The adhesion of Cu_2_O nanoparticles over cells membrane is observed on the TEM image (Fig. 6b) demonstrating interaction of Cu_2_O directly with the cell membrane, displaying shrunken cytoplasmic content and membrane detachment with associated rupture of cell wall. The cellular rupture and membrane disruption could be attributed to membrane binding and internalization of the Cu_2_O NPs. The Cu_2_O NPs and the cuprous ions released from the nanoparticles interact with the anionic bacterial cell surface leading to disequilibrium on the cell causing permeation with subsequent cell death. As displayed on the TEM image (Fig. 6b) the Cu_2_O NPs are strongly bound to the cell surface, the strong binding of the NPs could be attributed to electrostatic, covalent, Vander Waals forces causing membrane damage and leakage of intracellular components^9, 59^. After attachment on the bacterial surface the Cu_2_O NPs enter bacterial body and interact with basic components such as DNA, lysosomes, ribosomes and enzymes, leading to oxidative stress, heterogeneous alterations, changes in cell membrane permeability, electrolyte balance disorders, enzyme inhibition, protein deactivation, and changes in gene expression^13, 61^.

Besides mechanisms of physical interaction with the cellular machinery, the other plausible mechanism is through release of free cuprous ions (Cu^+1^) ,which cause membrane disruption through either strong electrostatic interaction between the positively charged Cu^+1^ ions and the negatively charged cellular membranes or via generation of intracellular reactive oxygen species^9, 59, 62^. Especially, Cu^+1^ ions have strong affinity for the amines and carboxyl groups present on the cell surface of the Gram-positive strains*,* which might explain their relatively higher antimicrobial activity against *B. subtilis* CN2 strain even if it is not statistically significant compared to the Gram-negative *P. aeruginosa* CB1 strain (*p* > 0.05). Previous studies reported CuO NP-associated toxicity to be predominantly mediated by dissolved Cu^2+^ ions than physicochemical properties of copper oxide nanoparticles^59^.

The other proposed mechanism of antibacterial action of the Cu_2_O NPs is ROS induced oxidative stress. ROS are comprised of “short-lived oxidants, such as superoxide radicals (O^−2^), hydrogen peroxide (H_2_O_2_), hydroxyl radicals (OH^−1^), and singlet oxygen (O^−2^)”^63^. ROS are normally generated under “physiological conditions, whereby the antioxidant machinery is enough to maintain equilibrium between production and scavenging of ROS, commonly known as redox homeostasis. However, when ROS production overwhelms the cellular scavenging capacity suspending cellular redox homeostasis, the results is a rapid and transient excess of ROS, known as oxidative stress”^64^. In the current study Cu_2_O NPs cytotoxicity as measured by the MTT assays revealed a significantly decreasing mitochondrial function with an increasing dosage of Cu_2_O NPs. The decreasing cell viability is supported by membrane disruption and lysis as observed on the TEM morphology micrographs. Based on cell viability and cell morphology assays and associated increase in intracellular ROS levels in the cells we can suggest that the dose dependent ROS induced oxidative stress is the other probable mechanism of antibacterial activity of the copper nanoparticles.

A significant increase in ROS and associated decrease in cell viability in a dose dependent manner, indicates that ROS could have contributed to cell membrane leakage and inflammation, resulting in cell-cycle arrest and subsequent cell death through generation of oxidative stress^65^. Our results are in accordance with previous studies that treatment of cells with Cu_2_O NPs can cause cytotoxicity and DNA damage to biomolecules such as DNA, proteins and lipids through generation of significant amount of ROS induced oxidative stress^52, 53^. The mechanism of nanoparticles induced oxidative stress varies among different nanoparticles and the underlying mechanism of ROS production is not clearly understood. Mechanism of nanoparticles induced oxidative stress may involve combination of generation of ROS on the metallic surface of nanoparticles, release of dissolved ions, and cell-NP physical interaction with subsequent alteration and rupture of membrane^66^. It is well known that free radical yielded by metals including copper cause radical mediated toxicity via Fenton-type reactions, while mitochondrial damage plays a major role in inert nanomaterials-based ROS generation^67^. Large quantities of Cu^+^ ions released from “Cu_2_O NPs both in the suspension and in cell medium, generate large amounts of OH by catalyzing Fenton reactions, leading to damage of lipids, proteins, and nucleic acids”^68^. Furthermore, oxidative stress leading to DNA damage may be caused by the intracellular Cu_2_O NPs that can directly interact with oxidative organelles such as mitochondria or attach to acidic components such as nucleic acid releasing more Cu^+^ from the Cu_2_O NPs^69^.

Generally, disintegration of membrane integrity succeeded by uncontrolled transport of Cu_2_O NPs and ultimate cell death sounds to have been caused by the joint action of adherence of the copper nanoparticles to the bacterial cells and generation of ROS. The nanometric scale surfactant stabilized Cu_2_O NPs demonstrated an enhanced antibacterial activity owing to their higher surface-to-volume ratio and increased number of atoms that interact with bacterial cell membrane, resulting in the formation of more ROS per unit weight, and higher probability to pass the cell membrane^41^. Furthermore, Cu_2_O NPs or ions dissolved from the nanoparticles may cause toxicity after entering the microorganisms’ body and causing depletion of intracellular ATP production and disruption of normal DNA replication^59^. The cuprous ions interact with biomolecules, such as “mercapto (–SH), amino (–NH), and carboxyl (–COOH) groups”, enzymes and lipids of the microbes after being slowly released from the nanoparticles and affect physiological process ultimately causing cellular death and inhibition of microbial growth^13^. Hence, we conclude that the toxicity is proposed to have been caused by the combined mechanism of ROS induced membrane damage and adhesion of Cu_2_O NPs on bacterial body causing increased membrane permeability, disruption and leakage of intracellular components.

Although clinical application of copper oxide nanoparticles is controversial due to potential adverse effects to human cells, several studies reported less or no toxicity of copper oxide nanoparticles^59, 70^. Unfortunately, the dependence of efficient bactericidal activity of Cu_2_O NPs up on the dissolved Cu^+1^ ions and solubility of the nanoparticles at lower pH significantly decreases their potential clinical applications at physiological pHs (6–8). Albeit, it is not the scope of the current study several studies have been conducted to exploit the enhanced antibacterial activity of copper oxide nanoparticles through readily releasing biocidal concentrations of copper ions at physiological pHs through synthesis of ligand modified copper oxo–hydroxide nanoparticles^71, 72^. The copper oxo-hydroxide nanoparticle modified with carboxylic acid ligands or tartaric/adipic acids demonstrated rapid release of copper ions in bacterial growth medium at physiological pHs^72^. Bastos et al.^71^, reported synthesis of copper oxo- hydroxide adipate tartrate (CHAT) that can release copper ions at effective antimicrobial level at pH 7.2 ± 0.2, demonstrating efficient antimicrobial activity.

## Conclusions

In the current study biocompatible and monodispersed, Cu_2_O NPs were synthesized using reverse micelle technique with environmentally benign microbial surfactant as a stabilizer. The lipopeptidal surfactant stabilized Cu_2_O NPs displayed a remarkable dose and pH dependent antibacterial activity against both Gram-negative and Gram-positive strains compared to the larger bare Cu_2_O NPs. The microbial surfactant stabilized copper nanoparticles with narrow size distribution showed a more effective contact biocidal and ion release property than bare nanoparticles. The smaller NPs larger surface area to volume ratio might greatly increase the production of ROS, which can damage and inactivate essential biomolecules compared to the bare Cu_2_O NPs that had shown extensive aggregation and a high degree of polydispersity with less antibacterial activity accordingly. The smaller sized and colloidal stable surfactant stabilized Cu_2_O NPs showed an enhanced antibacterial activity against both the Gram-negative and Gram-positive strains due to the higher surface area of the smaller nanoparticles for interaction with microbial bodies, increased solubilization of copper ions and the higher number of atoms interacting with the microbial membrane. The study highlighted that biocompatible Cu_2_O NPs might be developed with potential therapeutic applications, offering a promising solution to combat drug resistant bacteria which are becoming growing concerns globally, but lots of challenges still remain unanswered for the translation to clinical and actual applications. Despite the execution of multiple simultaneous bactericidal pathways to achieve antimicrobial activity, the mechanisms of antibacterial action of nanoparticles is still not clearly elucidated. Thus, future studies should be conducted to unravel the modes of action of the nanoparticles and investigate their biocompatibility for clinical applications through standardized nanotoxicology assays and protocols to assist easy comparison of data originating from in vitro and in vivo studies.

## Materials and methods

### Materials

Copper sulphate pentahydrate (> 99.8%), sodium borohydride _(_> 99.8%), Sodium hydroxide (> 99.8%), Tryptone soya broth (TSB; Difco), nutrient agar (Difco), Phosphate Buffered Saline (PBS, pH 7.4) were procured from Sigma Aldrich (St. Louis , MO, USA ). All chemicals used were analytical grade and utilized with no further purification. Deionized ultrapure water was used for dilution of chemicals throughout the study. Model Gram-negative *P. aeruginosa* CB1 and Gram-positive *B. subtilis* CN2 strains that were previously isolated in our lab are used in the study^73^.

### Synthesis and characterization of the cuprous oxide nanoparticles (Cu_2_O NPs)

Preparation of Cu_2_O nanoparticles by the reverse micelle technique was conducted with the microbial surfactant synthesized by *Bacillus cereus* SPL-4, identified as lipopeptide, as described in our previous study^74^, n-butanol as the co-surfactant, and n-heptane as oil phase. A typical synthesis of copper nanoparticles involved the mixing of two reverse microemulsions (microemulsion I and II). Microemulsion I consisted of 100 mL of dissolved lipopeptide solution (1 g/L, 2 g/L), 25 mL of n-butanol, 25 mL of n-heptane, 100 mL of 0.3 mol/L CuSO_4_.5H_2_O solution. Microemulsion II contained 50 mL of 1.6 mol/L NaBH_4_ aqueous solution added to the same amount of lipopeptide solution, n-butanol and n-heptane. A solution of NaOH (1 M) was used to adjust the pH of Microemulsion I up to 12. After stirring at room temperature for about 10 min, Microemulsion II was added dropwise to microemulsion I at room temperature under vigorous mechanical stirring for 30 min. The formation of the nanoparticles was accompanied with a colour change from blue colour of the reaction mixture to darker and eventually light-red. Afterwards, the solution was aged for an hour and the nanoparticles formed in the reverse micelles were collected by centrifugation (12,000 rpm, 4 °C for 10 min), washed three times with water and ethanol mixture (1:1) to get rid of the remaining surfactants and other organic residuals and left to dry in vacuum drier at 50 °C for 5 h. The fabricated nanoparticles were left in an open air for 24 h to dispose of the residual unreacted sodium borohydride reductant before antibacterial activity study was conducted.

The crystal structure and phases present in the as-synthesised samples were analysed using X-ray powder diffraction (XRD) using a PANalytical X’Pert Pro powder diffractometer in θ–θ configuration with an X’Celerator detector and variable divergence- and fixed receiving slits with Fe filtered Co-Kα radiation (λ = 1.789 Å). The mineralogy was determined by selecting the best–fitting pattern from the ICSD database to the measured diffraction pattern, using X’Pert Highscore plus software. The relative phase amounts (wt%) were estimated using the Rietveld method (X’Pert Highscore Software).

The size, size distribution, morphologies and composition of the samples were visualized by transmission electron microscopy (TEM) on a JOEL JEM-2100F transmission electron microscope, and the acceleration voltage was 200 kV and UV–Vis spectroscopy. High speed elemental analysis of the as synthesised nanoparticles was carried out using transmission electron microscopy energy dispersive X-ray spectroscopy (TEM-EDS). Ultrapure water diluted nanoparticle suspension was sonicated for 10 min, spread on copper grid, dried overnight and TEM analysed. The UV–Vis spectra of the nanoparticles were recorded using UV–Vis spectrophotometer after dispersion of the nanoparticles in ultrapure water (10 mg/L). The surface morphology of the as-prepared nanoparticles was further characterized by a high-resolution Zeiss Ultra Plus 55 field emission scanning electron microscopes (FE-SEM) operated at 2.0 kV. A thinly layer of nanoparticle powder was spread on SEM stub mount and covered with a ~ 10 nm carbon film for the analysis.

### Test of antibacterial activity of the fabricated Cu_2_O NPs

For reliable antibacterial activity assessment of the Cu_2_O NPs, all equipment and materials used were sterilized by autoclaving at 121 °C for 15 min. The two selected strains for the study were *P. aeruginosa* CB1 and *B. subtilis* CN2, which are typical representatives of Gram-negative and Gram-positive multidrug resistant bacterial strains respectively, previously isolated in our lab^73^.

### Testing the antimicrobial effect by minimum inhibitory concentration

The antimicrobial activity of the Cu_2_O NPs in terms of the minimum inhibitory concentration (MIC) were tested by the modified broth macro dilution technique of Clinical and Laboratory Standards Institute (CLSI)^75^. Stock solution of Cu_2_O NPs at 1000 μg/mL are freshly prepared and serial dilutions of Cu_2_O NPs (500, 250, 125, 62.5, 31.25, 15.625, and 0 μg/mL) were prepared in Erlenmeyer flasks using tryptone soya broth (TSB) (Oxoid). The bacterial strains for inoculation were grown overnight in tryptone soya broth at 37 °C, 150 rpm aeration and harvested by centrifugation (10,000 rpm, 4 °C for 10 min). The cell pellets were washed twice in phosphate buffered saline solution (PBS, pH 7.4), resuspended and diluted in the PBS solution to an OD at 600 nm (OD_600_) of 0.5, corresponding to approximately 10^9^ CFU/mL. Flasks containing nutrient broth with varying concentrations of Cu_2_O NPs were inoculated with 1 mL of bacterial suspension and incubated for 24 h at 37 °C and 150 rpm. Bacterial growth was monitored by measuring optical density (OD_600_) using UV–Vis spectrophotometer. Minimum inhibitory concentration (MIC), the lowest concentration of the antimicrobial agent preventing visible growth of each microorganism, was determined after incubation. From the results obtained for growth inhibition, IC_50_ (concentrations at which 50% of bacterial proliferation are inhibited) are determined. All experiments were done in triplicates and results are reported as mean ± SD and flasks with no Cu_2_O NPs were used as positive control. Excel linear regression analysis was carried out to analyse cell viability.

### Agar well plate diffusion method

The antibacterial activities of Cu_2_O NPs were investigated by agar well plate diffusion method according to the guidelines of the National Committee for Clinical Laboratory Standards^76^. The tested microorganisms were spread evenly on nutrient agar plates using a sterile loop, then wells of 6-mm diameter were made using sterile well borer, and Cu_2_O NPs with concentrations of 1 mg/mL and 2 mg/mL were added. The average diameter of zone of inhibition surrounding the wells was measured to the nearest 0.5 mm resolution with a ruler after incubation of the plates for 24 h at 37 °C. The mean and standard deviations reported for the Cu_2_O NPs with each microbial strain were based on triplicates.

### Measurement of cell viability

Cellular viability after Cu_2_O NPs treatment was assessed following the method used by Sahoo et al.^54^. This assay is based on the reduction of MTT (3-(4,5-dimethylthiazol-2-yl)-2,5-diphenyltetrazolium) to a dark blue hydrophobic formazan product by mitochondrial dehydrogenase enzyme. Cells of approximate size 10 ^6^ CFU’s/well were subjected to treatment of Cu_2_O NPs at different concentrations for 24 h at 37 °C. After incubation for 24 h the medium was removed and replaced by new 100 μL medium and 20 μL of MTT (5 mg/mL in PBS) and incubated for 4 h at 37 °C. Subsequently the resulting formazan product was dissolved in DMSO (100 µL) and the absorbance intensity measured by a microplate reader (Synergy-HT, BioTek, Virginia, USA) at 570 nm. All experiments were run in triplicates and cell viability was expressed as a percentage relative to the untreated control cells.

### Estimation of reactive oxygen species generation (ROS)

Intracellular ROS generated in cells following Cu_2_O NPs treatment was analysed using the fluorescent probe 2′,7′-di-chlorofluorescin diacetate (DCFDA), a non-fluorescent compound under normal condition. With subsequent internalization of DCFDA by the cells, cellular esterase mediated hydrolysis of the dye takes place to a non-fluorescent compound, which later is oxidized by ROS to a highly fluorescent 2′7′-dichlorofluorescein (DCF) compound that can be detected using fluorescence spectroscopy. Briefly, the cells were grown overnight at 37 °C , harvested by centrifugation (3500 rpm for 5 min) and treated with different concentrations of Cu_2_O NPs for 24 h at 37 °C. After 24 h of treatment, cells were harvested by centrifugation at 3500 rpm for 5 min at 4 °C, washed three times with PBS and incubated with 1000 μL of 25 μM of DCFH-DA for 30 min at 37 °C in the dark. Afterwards, cells were harvested and washed with PBS and analysed by flow cytometry (Accuri C6 Plus flow cytometer, BD Biosciences). Untreated samples were included as a negative control and the data were analysed using BD Accuri C6 software.

Intracellular ROS localization was determined using confocal microscopy, following fluorescent probe 2′,7′-dichlorofluorescein diacetate (DCFDA) probe. After 24 h of treatment, cells were harvested by centrifugation at 3500 rpm for 5 min at 4 °C, washed three times with PBS and incubated with 1000 μL of 25 μM of DCFH-DA for 30 min at 37 °C in the dark. Afterwards, cells were harvested and washed with PBS and observed with Zeiss Confocal Laser Scanning Microscope 880 with excitation at 488 and emission at 515 to 530 nm.

### Transmission electron microscopy (TEM) and scanning electron microscopy (SEM) observation of Cu_2_O NPs treated cells

Morphological change of cells after exposure to the Cu_2_O NPs was observed using Transmission electron microscopy (TEM) and scanning electron microscopy (SEM). The cells were grown overnight at 37 °C in 5% CO_2_, harvested by centrifugation (3500 rpm, 4 °C, for 5 min) and treated with different concentrations of Cu_2_O NPs for 24 h at 37 °C in 5% CO_2_. Subsequently, the pellets we collected by centrifugation (3500 rpm, 4 °C, for 5 min), washed with phosphate buffered saline solution trice and fixed with 2.5% glutaraldehyde and 2.5% formaldehyde for 1 h, washed trice with PBS, and postfixed with 1% osmium tetroxide for 1 h.

After fixation, the specimens were dehydrated in increasing concentration of ethanol (30%, 50%, 70%, 90%, and 3 × 100%, respectively), and embedded in 100% epoxy resin and left to polymerize at 55 °C in 5% CO_2_ for 36 h. The resin blocks were then sectioned using an ultramicrotome. The untrathine sections of bacterial cells were placed on the grids, stained with uranyl acetate and lead citrate solution for TEM observation (JOEL JEM 2100F).

For SEM observation, after 24 h treatment the specimens were postfixed with 2.5% glutaraldehyde for 1 h, washed 3 times with PBS (pH 7.4), fixed with 1% osmium tetroxide for 1 h, dehydrated with increasing concentration of ethanol (30%, 50%, 70%, 90%, and 3 × 100%, respectively). Then the specimens were chemical dried with an increasing concentration of hexamethyldisalzane succeeded by overnight air drying, sputter coated with 15 nm platinum and then observed using a Field Emission Scanning Electron Microscope (FE-SEM) Zeiss ULTRA Plus (Germany).

### Assessment of dissolution and cellular uptake of Cu_2_O NPs

Quantitative evaluation of dissolution of Cu^+^ ions and cellular uptake of Cu^+^ and Cu_2_O NPs was performed according to the method described by Ahmed et al.^77^. To determine Cu^+^ ion dissolution, the cells were grown overnight at 37 °C in 5% CO_2_ incubator, harvested by centrifugation (3500 rpm, 4 °C for 5 min) and treated with different concentrations of Cu_2_O NPs for 24 h at 37 °C in 5% CO_2_ incubator. After 24 h exposure the supernatant was collected by centrifugation (12,000 rpm, 4 °C, 10 min), filtered through 0.22 µm membrane filter and the Cu^+^ concentration was determined by AAS, (Perkin Elmer analyst 400 AAS). Similarly, Cu^+^ ions and internalized Cu_2_O NPs in the bacterial cells was determined after treatment of the Gram-positive and Gram-negative isolates with different concentrations of the Cu_2_O NPs (62.5, 125, 250 µg/mL) at pH 7 and pH 5 for 24 h at 37 °C. Cells were counted, the cell pellet was collected by centrifugation (10,000 rpm, 4 °C, for 10 min), washed three times with PBS to remove the adsorbed Cu_2_O NPs, digested with 3 mL of fresh aqua regia for 12 h and then diluted to a total volume of 10 mL with Milli-Q water. The concentration of internalized copper was measured by AAS (Perkin Elmer analyst 400 AAS) and reported as the mass of copper per cell.

### Statistical analysis

Statistical software IBM SPSS Statistics 23.0 (SPSS Inc., Chicago, IL, USA) was used to evaluate statistical significance of the treatments. One-way analysis of variance (*ANOVA*) was applied to evaluate differences between treatments. The outcomes were considered statistically significant compared with the control when *p* values are < 0.05.

## Supplementary information

Supplementary Legends.

Supplementary Figure S1.

Supplementary Figure S2.

## Data Availability

DNA sequence of the strains *Pseudomonas aeruginosa* CB1 and *Bacillus subtilis* CN2 are deposited in the GenBank database under the accession numbers KP793922 and KP793926 respectively.
