# Supplementary material for: Fabrication of monodispersed copper oxide nanoparticles with potential application as antimicrobial agents
Source: Sci Rep. 2020 Oct 7;10:16680. doi: 10.1038/s41598-020-73497-z (PMC7541485; doi:10.1038/s41598-020-73497-z)
Supplement: Supplementary file 1 — Supplementary Legends. [file 41598_2020_73497_MOESM1_ESM.docx]

**Supplementary Figure S1.** Zone of inhibition of lipopeptide surfactant stabilized Cu_2_O NPs against (a) *B. subtilis* CN2 and (b) P. *aeruginosa* CB1 strains

**Supplementary Figure S2**. Oxidative stress response at various doses of Cu_2_O NPs on the Gram-negative *P. aeruginosa* CB1 (left) and Gram-positive *B. subtilis* CN2 (right) bacterial cells. (a,b) Control cells; (c, d) Cells treated with 50 µg/mL; (e, f) cells treated with 100 µg/mL; (g, h) cells treated with 125 µg/mL for 24 h. After treatment of cells with designated concentrations of Cu_2_O NPs for 24 h, intracellular ROS generation was quantified by oxidation of cell permeable dye 2,7-dichlorodihydrofluorescein diacetate (DCFDA) staining using flow cytometer.
