# Supplementary material for: Fabrication of monodispersed copper oxide nanoparticles with potential application as antimicrobial agents
Source: Sci Rep. 2020 Oct 7;10:16680. doi: 10.1038/s41598-020-73497-z (PMC7541485; doi:10.1038/s41598-020-73497-z)

**Fabrication of Monodispersed Copper Oxide Nanoparticles with Potential Application as Antimicrobial Agents**

Fisseha A Bezza^a*^, Shepherd M Tichapondwa^a^, Evans MN Chirwa^a^

^a^ Water Utilization and Environmental Engineering Division, Department of Chemical Engineering, University of Pretoria, Pretoria 0002, South Africa

[*fissehaandualem@gmail.com](mailto:*fissehaandualem@gmail.com)


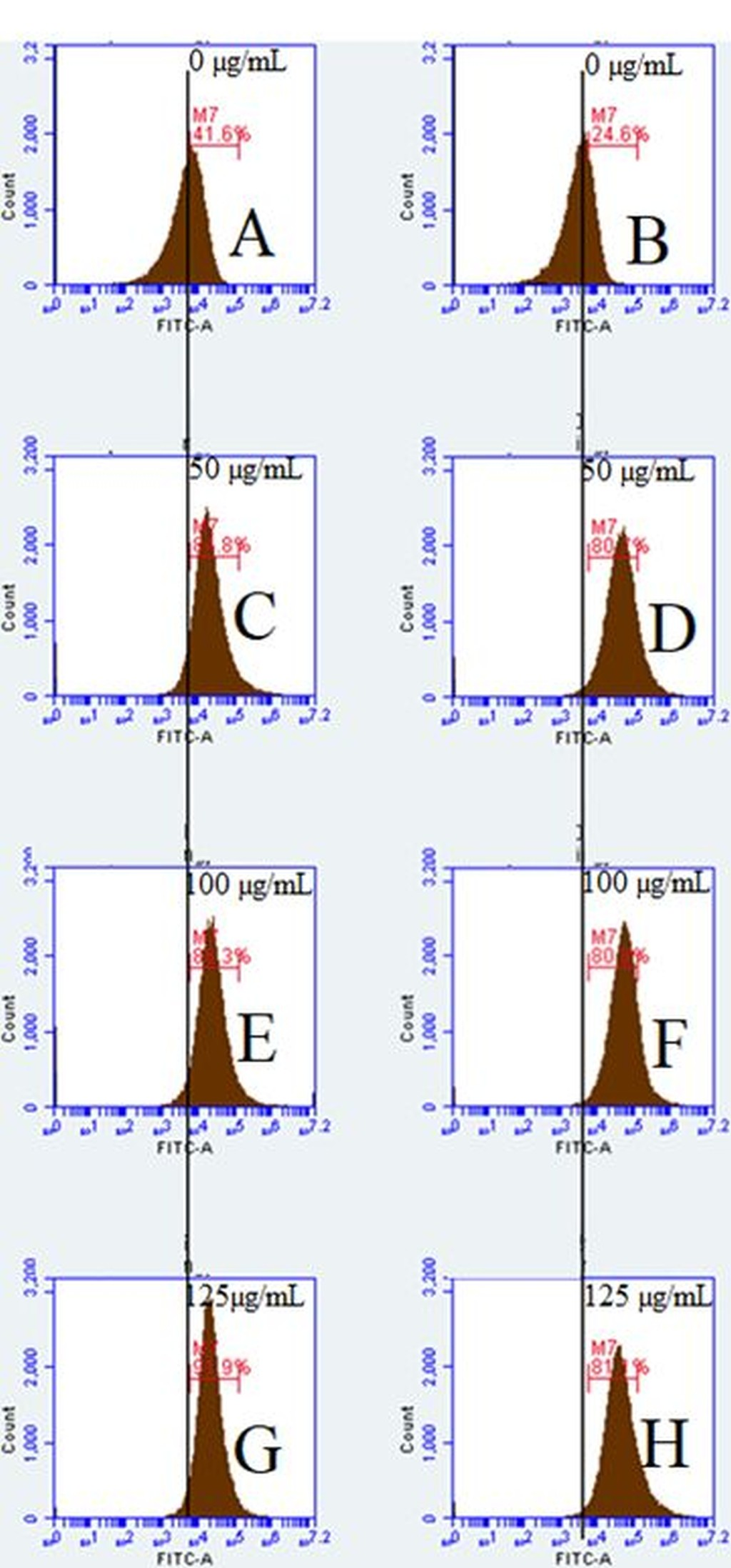


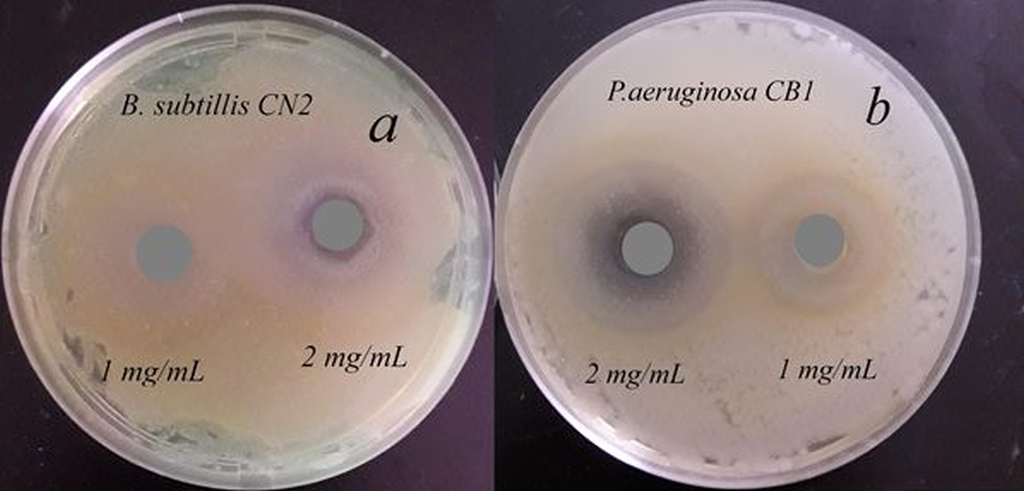

Supplement: Supplementary file 3 — Supplementary Figure S2. [file 41598_2020_73497_MOESM3_ESM.docx]
